# Progress in palliative care for cancer in Turkey: a review of the literature

**DOI:** 10.3332/ecancer.2021.1321

**Published:** 2021-11-25

**Authors:** Tezer Kutluk, Fahad Ahmed, Mustafa Cemaloğlu, Burça Aydın, Meltem Şengelen, Meral Kirazli, Sema Yurduşen, Richard Sullivan, Richard Harding

**Affiliations:** 1 Department of Pediatric Oncology, Hacettepe University Faculty of Medicine and Cancer Institute, 06100 Ankara, Turkey; 2 Department of Public Health, Hacettepe University Faculty of Medicine, 06100 Ankara, Turkey; 3 King’s College London, Institute of Cancer Policy, Conflict & Health Research Group, London, UK; 4 Florence Nightingale Faculty of Nursing, Midwifery and Palliative Care, Cicely Saunders Institute, King’s College London, London, UK

**Keywords:** palliative care, cancer, capacity building, systematic review, Turkey

## Abstract

**Background:**

The demographic transition in Turkey is shifting the burden of diseases towards non-communicable diseases including cancer. Palliative care (PC) as a component of Universal Health Coverage assures patient and family-centred care provision throughout the spectrum of cancer.

**Objectives:**

This study aimed to make a detailed evaluation of the progress achieved since the mid-90s and the current situation of cancer PC in Turkey.

**Methods:**

A literature review was conducted in PubMed, Scopus, Embase, ScienceDirect, Web of Science, Google Scholar, The Turkish Academic Network and Information Centre databases, Ministry of Health documents, Council of Higher Education’s thesis 01/1995 to 07/2020. The information was categorised into the six domains: history of the cancer PC; law and regulations; education and research; opioid use; patient care and palliative centres; public awareness, psychosocial support and end of life ethics.

**Results:**

Of 27,489 studies, 331 met the inclusion criteria. The majority were published in the Turkish language and were journal articles. The findings showed that the development of PC in Turkey can be divided into three stages: early initiatives before 2000, the dissemination stage, 2000–2010 and the advanced stage after 2010. There is evidence of progress in terms of legal regulations, opioid use and number of PC services and research output. However, there is still a need for improvement in professional education, public awareness and end of life care.

**Conclusion:**

There is evidence of progress, barriers and opportunities. However, bringing research into practice is needed for scale-up and integration of PC in cancer care in Turkey.

## Background

Demographic and disease transition has amplified the challenges for cancer care globally [1–3]. Palliative care (PC) aims to relieve the suffering of patients facing life-limiting conditions. Although PC is a newer component in the modern healthcare system, it is increasingly recognised as an essential part of it. The PC resolution adopted at the World Health Assembly in May 2014, urged governments to ‘integrate palliative care services in the continuum of care, across all levels, with emphasis on primary care, community and home-base care, and universal health coverage (UHC) schemes’ [4]. Moreover, the recommendations of the Worldwide Palliative Care Alliance stress that all governments must integrate PC along with preventive and curative health care into their national health system [5].

It is estimated that more than 56.8 million people need end of life PC every year globally [5]**.** If it is considered that for every terminally ill patient requiring PC, there are at least one or two caregivers involved, the total need of PC will be twice or thrice than the above estimates. Owing to physical symptoms, psycho-social and treatment effects, cancer patients are in greater need of PC and it is estimated that cancer patients only require more than one-quarter of global PC need [5]**.**

Considering the need of PC, it is also worth noting that only a few countries have comprehensive PC programmes through a public health approach [6]. According to the most recent global survey conducted among 194 countries, funding for PC was available in 68% of countries and only 40% of countries reported that the PC services reached at least half of the patients in need [7].

Turkey is home to about 83.6 million people [8] and additionally hosts about 3.6 million refugees [9]. Based on the Global Cancer Observatory (GLOBOCAN) 2020 data, the estimated number of annual cancer cases in Turkey is 233,834 and 126,335 people die due to cancer [10]. Considering the burden of cancer and other non-communicable diseases (NCDs) along with an increase in life expectancy at birth [11], the real need for PC will be much greater in the near future. The concept of PC was developed in Turkey along with the cancer control programme. The Turkish Ministry of Health (MoH) had brought together all stakeholders under the umbrella of a National Cancer Advisory Board and ignited the Palliaturk project which became a turning point to create a national policy for PC [12]. Despite the decade long effort for establishment of the national PC programme and the progress which has been made, it is widely acknowledged that there is still a need for improvement for further dissemination and to take PC to a higher level in Turkey [13]. The aim of this review is to make a detailed evaluation of the progress achieved since the mid-90s and the current situation of cancer PC in Turkey.

## Method

### Search strategy

The methods for this review were based on Arksey and O’Malley’s scoping review methodology [14]. The databases were retrieved through a search of MEDLINE, Scopus, Embase, ScienceDirect and Web of Science, Google Scholar, ULAKBIM (The Turkish Academic Network and Information Centre), Turkish MoH documents, The Thesis Database of the Turkish Council of Higher Education and renowned national and international PC as well as cancer conferences. Articles were explored for PC for cancer patients in Turkey. The search was made by using three Medical Subject Headings (MeSH) categories – palliative care (palliative care, palliative therapy, end of life care, terminal care, supportive care, palliative medicine), cancer (cancer, oncology, malignancy, neoplasm, tumours, neoplasia) and Turkey (Turkey, Turkish) – combined using ‘and’ statements. The local database was also searched using the translation of the above term in Turkish. The search was limited to literature published between the year 01/1995 and 07/2020. Searches were performed on article titles, abstracts and full text. Additional studies were identified through the references of relevant studies.

### Data selection procedure

Articles/studies were included in the review if they were in either the English or Turkish language and focused on PC in cancer. Articles which did not focus on Turkey, or were focused on surgical or radiological interventions for palliation of symptoms were excluded. Following the database search, duplicates were removed. Titles and abstracts were inspected jointly by all authors for inclusion in the review. Irrelevant studies were removed and the full text was examined if necessary. A Preferred Reporting Items for Systematic Reviews and Meta-Analyses (PRISMA) flow diagram was used to provide information regarding the selection of the articles in this review.

### Data extraction and analysis

The following information was extracted from articles: author names, the institution of affiliation of the first and corresponding author, title, name of the journal/conference/publisher, year of publication, type of publication, language of the article, study design and setting, national or international collaboration, aim/objective of the study, aspect of PC being studied and the main findings.

The main text of all articles included in this review was read by authors independently, the key findings were extracted and assigned into categories. Later these categories were examined and conceptualised in the research team meetings and were condensed into major themes; history of PC in Turkey, law and regulations, education & training, research trend, pain management, patient care and psychosocial support. The summary findings are presented in the results section.

## Results

### Search results

**Figure 1.**
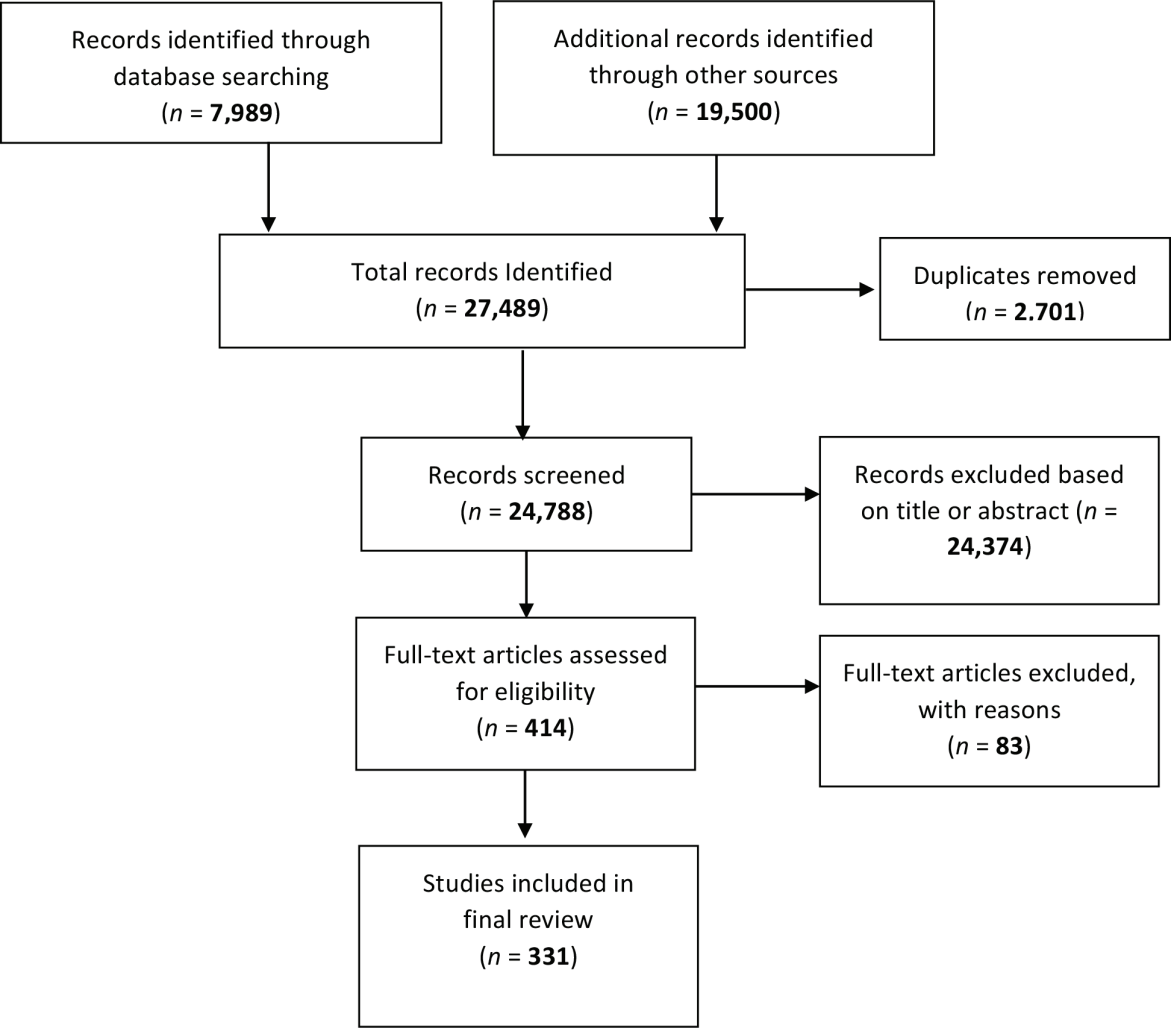
PRISMA flowchart.

A total of 27,489 papers (7,989 research papers, 19,388 abstracts of conference presentation/poster, 101 postgraduate theses and 11 governmental documents) were identified. After removing duplicates, 24,788 papers met the condition for full-text screening. In all, 24,374 pieces of literature (the large majority of conference abstracts) were excluded based on the title or abstract. The remaining 414 were retrieved and screened in detail. Out of these, 83 were removed (there was insufficient information regarding PC for cancer in the full text of 67 literatures, five studies were specific to ethical considerations at the end of life, four were about the specific surgical procedures to palliate the obstructive symptoms due to cancer, other four were regarding PC needs of neonates with congenital malformation/birth defects, two studies were regarding geriatric health without cancer, one was about burnout of healthcare workers at oncology services). Figure 1 describes the PRISMA flowchart of the literature reviewed in this study and the details of the included 331 articles are shown in Supplementary Table 1.

### Characteristics of included studies

Of these 331 articles included in this review, the majority 161 (48.6%) are journal articles, 96 (29.0%) conference abstracts, 56 (16.9%) post-grad theses, 13 (3.9%) gray literature (mostly from the Turkish Ministry of Health), 3 (0.9%) book chapters and 2 (0.6%) are letter to a journal editor. Regarding journal articles – more than half, 98, articles were published in international journals, whereas 63 were published in Turkish/national journals.

**Figure 2.**
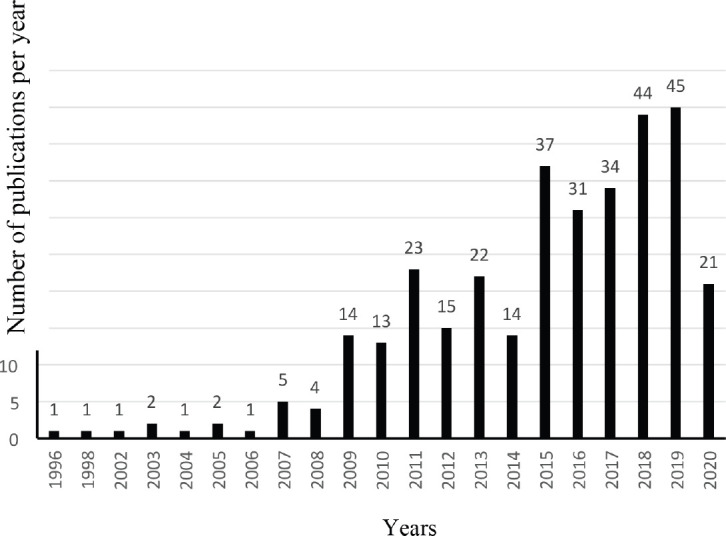
The evolution of the number of articles regarding PC for cancer in Turkey published per year, 1996–2020.

About 193 (58.3%) literatures were published in the Turkish language, whereas 138 (41.7%) were in English. Among all the literature included in this review, 194 (58.6%) used cross-sectional surveys or retrospective analysis of datasets, 59 (17.8%) were review articles, letter to the editors, book chapters, 19 (5.7%) employed either interventional design or randomised control trials or quasi-experimental design, 14 (4.2%) studies evaluated validity and reliability of specific questionnaires among Turkish PC cancer patients, 13 (3.9%) were gray literature, 12 (3.6%) used qualitative research methods, 5 (1.5%) were case studies or case series evaluating a specific dimension of PC for cancer patients, 4 (1.2%) employed methodological design, 3 (0.9%) mixed methods research design, 3 (0.9%) articles used either prospective or cohort design and other 3 (0.9%) were cost analysis studies. Only 2 (0.6%) used a case–control design. Furthermore, 11 articles were published in collaboration with the Middle East Cancer Consortium (MECC). As shown in Figure 2, only two articles included in this review were published before the year 2000. Most of the articles were published between the year 2018 and 2019. The major themes were presented below.

### History of cancer PC in Turkey

**Figure 3.**
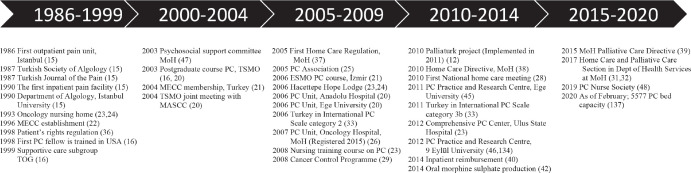
The evolution of the PC in Turkey from the mid-1980s to 2020. PC, Palliative care; MECC, Middle East Cancer Consortium; MASCC, Multinational Association of Supportive Care in Cancer; MoH, Ministry of Health; TOG, Turkish Oncology Group; TSMO, Turkish Society of Medical Oncology; ESMO, European Society of Medical Oncology.

The history of PC in cancer is evaluated in three stages as summarised below and also shown in a timeline (Figure 3).

**The first stage: Early initiatives (Before 2000):** The earliest available information regarding PC is the establishment of the first outpatient pain unit in Istanbul University in 1986, and the establishment of the Turkish Society of Algology and the launch of the Turkish Journal of Pain in 1987. Soon after, in 1990, an inpatient pain department was established in Istanbul University and in the same year, algology was accepted as a speciality by Higher Education Council [15].

By the end of the 90s, there were no modern PC services. A lack of trained health care professionals, low public/professional awareness, limited access to opioids and opiophobia were the major barriers [16, 17]. In 2000, 13.1% of all deaths in Turkey were due to cancer and future projections showed that it would continue to rise [18]. Generally, PC was not a priority. By 2005, there were only seven supportive care units for pain and symptom management. Opioid consumption was relatively low and physicians as well as pharmacists faced legislative and practical obstacles in prescribing and dispensing opioid analgesics [19].

**The second stage: Dissemination of PC concept and awareness among the medical and scientific community (2000–2010):** The new millennium witnessed interest from governmental and non-governmental stakeholders. The Turkish Oncology Group (TOG) established a supportive care working group in 1999 [16]. The National Cancer Advisory Board of MoH sets a psychosocial sub-committee in 2003. The Turkish Society of Medical Oncology started training courses [16, 20], organised a joint meeting with the Multinational Association of Supportive Care in Cancer (MASCC) in 2004 [20] and the European Society for Medical Oncology (ESMO) in 2006 [21]. The MECC, founded in 1996 in collaboration with MoH from member countries, became a platform bringing together clinical professionals and researchers [22]. As soon as Turkey became a member of MECC in 2004 [21], MECC & MoH collaboration started to organise meetings in cancer control including PC courses and workshops. PC awareness and engagement of different stakeholders were raised in the mid-2000s; The establishments of Hope Lodge by Hacettepe University Oncology Institute Foundation in 2006 [23, 24], the Palliative Care Association (PCA) in 2005 [23, 25], PC units in Ege University and Anadolu Hospital in 2006 [20] and a PC unit at MoH Oncology Hospital in 2007 [26] are examples of progress in this period. Based on a survey by MoH in 2009, there were only 10 PC units and 72 pain units in the country. Morphine use was less than in the USA and many Middle Eastern countries. Legal restrictions and lack of trained PC staff were the major barriers [12, 27, 28].

**The third stage: Advancement with government and societal engagement (After 2010):** MoH brought the first Cancer Control Programme in 2008 [29]. Soon after, MoH Cancer Control Department started the Palliaturk project in 2010, which was implemented in 2011 [12]. The Palliaturk project had two main objectives – namely targeting the availability of opioids and implementation of a community based PC model [12, 28]. The model included primary, secondary and tertiary level PC centres. MoH also started collaborations with international and national stakeholders (World Health Organization (WHO), Union for International Cancer Control (UICC), national non-governmental organisations (NGOs) and professional organisations, etc.) [12, 28]. Since the 2010s, new regulations and rules were set by the government; stakeholder involvement and awareness of the medical community as well as the public had increased. New PC centres were opened around the country. In 2015, the ‘Palliative Care Nursing Certificate Program’ was started by the MoH [30]. In 2017, The Home and Palliative Care section was established under MoH Public Health Directorate [31, 32]. According to the international PC scale ranking, Turkey was in group 2 (capacity-building PC activity) in 2006, and moved up to group 3b (generalised PC provision) in 2011, then in group 3a (isolated PC provision) in 2017. There were no countries except Israel in group 4 from the Middle Eastern Countries [6, 33–35].

### Law and regulations

The major PC regulations and laws were released after 1998. The first regulations relating to patient’s rights were released in 1998 [36]. The first home care regulation was released in 2005 [37]. PC services were mentioned in the 2010 update of the home care directive [20, 24, 38]. The first National Cancer Control Programme was released in 2008 [29] and became a turning point – the MoH Cancer Control Department started to work on PC Projects. With the aim of PC provisions, MoH established the PC Directive in 2014 and implemented it in 2015. It became a strong legal support to the establishment of PC centres to provide and promote PC in Turkey [39]. The PC directive described inpatient PC centres within the established hospitals. The involvement of family physicians and home care services in the outpatient setting were also included. The PC coverage includes examination, evaluation, care, rehabilitation, psychosocial support, nutritional support, pain management, legal support for the patients & relatives. Social Security Administration accepted the reimbursement of the inpatient PC costs in 2014 [20, 40]. An update of the home care regulation was released in 2015 [41]. In parallel to this legal progress, the first morphine sulphate tablet was officially produced by a Turkish pharmaceutical company in 2014 [42].

### Professional education, training and research

The first PC fellow of Turkey was a medical oncologist trained in the USA in 1997–1998. Between 2001 and 2010, the Supportive Care in Cancer Committee of the TOG organised 18 national/international training meetings. This committee also contributed to the development of PC within the National Cancer Control Programme after 2008 [16]. Turkish Medical Oncology Society organised the first postgraduate education on PC in 2003, joint meeting with MASCC in 2004, PC course with ESMO in 2006 [20]. More PC courses and meetings have been organised after 2013 by different stakeholders [20]. A nursing academic was trained in the USA for PC in 2006 [20]. MECC & MoH collaborations became a driving force for PC education in Turkey and regional countries. They jointly organised meetings and workshops between 2004 and 2014, training 434 health care professionals [20–22, 43, 44].

The interest in PC research and education from Medical & Nursing Schools appeared after 2010. PC lectures were included in Nursing schools under the postgraduate curriculum [20]. PC centres were established in Ege and Dokuz Eylul Universities in 2011 and 2012, respectively. The centre in Dokuz Eylul University was approved by the Higher Education Council [24, 45, 46].

Stakeholders including the Turkish Medical Oncology Society, the anaesthesiology/algology, nursing community and PCA made a significant contribution. The inclusion of a Psychosocial Committee within the National Cancer Advisory Board in 2003 also helped to increase stakeholders’ engagement [47]. In 2019, the Palliative Care Nursing Association was established [48]. The increasing number of reports from various stakeholders including medical specialities [49–51] nurses [20, 52–57], physiotherapists [58–62], social workers [47, 63], hospital managers [64], nursing students [65–68] shows the progress in the PC field in Turkey. However, there were an insufficient number of PC healthcare workers. The lack of PC training among medical, nursing, midwifery, students, physicians and nurses, healthcare staff, emergency care staff was within the range of 50%–80% [69–83].

The number of research outputs on PC increased after 2015. The different areas of PC were investigated. The need assessment and adaptation of the ENABLE (Educate, Nurture, Advise, Before Life Ends) evidence-based early PC model were also investigated for Turkish family caregivers of older persons with cancer [84]. Ulus State Hospital, MoH first comprehensive PC centre, reported that 38.4% PC patients had a cancer diagnosis [85]. A previous study found that pain was the most common symptom (27.1%) among hospitalized patients [86]. Along with an increase in PC services, burnout was also found to be a significant problem among healthcare workers working in PC units [87]. Complementary and alternative medicine (CAM) use is also a common practice in Turkey. CAM use was found to be 57% among cancer patients [88]. In another study, at least one CAM method was used by 62% [89]. Irmak *et al* [90] found that 46.4% of cancer patients were CAM users, however no significant difference was found with respect to quality-of-life (QoL) score among CAM users and non-users. A randomised trial found that listening to music was effective in controlling pain and anxiety among cancer patients [91]. Several studies focused on the validity, reliability and adaptation of various scales among PC and cancer patients [92–119] (See Supplementary Table 2).

### Opioid use and pain management

Opioid consumption at the global level started to increase in the mid-90s. A survey on the availability and accessibility of opioids among Middle East countries showed that opioid availability was low throughout most of the Middle East countries except Israel. In 2011, Turkey was in the 10th rank as regards morphine consumption mg per capita [120]. For many years, opioids are available but the process for prescribing was complicated with a colour-coded system; red prescription for strong opioids, green for sedatives and weak opioids and white for all non-restricted prescription [16].

Opioid consumption in total morphine equivalence, milligrams per capita, in Turkey in 1980 was 0.0937 and it increased to 12.2204 in 2011. Although there was a significant increase by the time, 2011 Eastern Mediterranean Regional Office (EMRO) average was 10.56, global average was 61.66 [34, 43]. A study published in 2010 showed that the consumption of morphine has been fluctuating at doses around 0.1 mg/capita in Turkey for the period of 2004–2007. This was higher than Saudi Arabia and Egypt but lower than Israel, Cyprus, Jordan and Lebanon. Morphine consumption in Turkey was 447 times less than the USA in 2007. In the USA, the consumption was 76 mg/capita in the year 2007. The global mean was 5.57 mg/capita [121]. There was a big disparity among the Middle East countries including Turkey in terms of opioid use compared with western World and the USA. Turkey was at number 50th in the world for consumption of opioids analgesics during 2007–2009 [22]. Access to Opioid Medicine in a European Project (ATOME) in 2016 reported that Turkey had more than 40 potential barriers in different categories [122].

A number of studies have been published with a focus on pain management in cancer patients [123–132]. A study among 52 metastatic cancer patients found that the use of morphine was 5.7%; codeine 3.8% and tramadol 75% [130]. In another study, interventional procedure was used to control pain in 11% of cancer patients [131]. It was also reported that 82% of hospitalised cancer patients for PC were given different analgesic treatment of whom 50% received third step pain medicines [124]. A study among 1,467 cancer patients utilising analgesic step ladder approach showed the use of nonopioid+/−weak opioid+strong opioid in 31.5% and interventional procedures in 14.5% of patients [125].

### Patient care and PC centres

PC activity was very limited in Turkey before 2010 [21]. The European Association for Palliative Care (EAPC) survey in 2007 showed that PC services are provided in ten centres with a total of 241 beds, and there was only one hospice centre in Turkey. Most of the PC centres were within oncology clinics with a major focus on pain control [133]. The earliest PC units were established in 2006 at Anadolu and Ege University Hospitals [20]. MoH Abdurrahman Yurtaslan Hospital established the comprehensive PC services in 2007 with 18 inpatient beds [26]. New PC centres in MoH Hospitals were established following 2010 [20]. A multidisciplinary PC team in 2010 and first PC unit for children in 2011 were established in Dokuz Eylul University [134, 135]. The first ‘Comprehensive PC Center’ in the Turkish MoH, Ulus State Hospital was opened in December 2012. One third of patients treated in this centre within a year had a cancer diagnosis [23].

**Figure 4.**
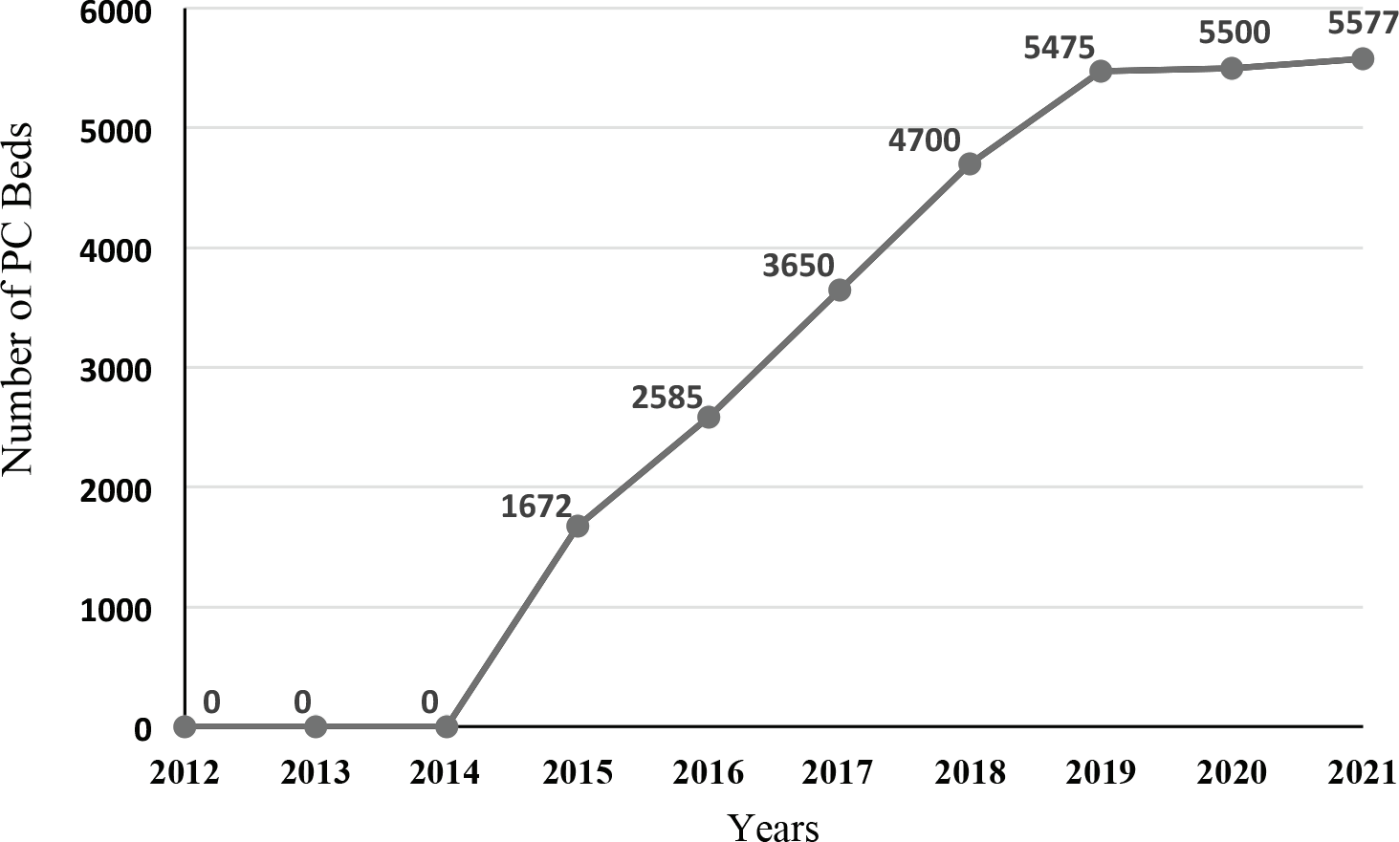
Number of PC beds in Turkey, 2012–2021.

PC services were included in home care services in 2010 [20, 38]. By the year 2014, there were a total of 834 home care teams providing services to 416,175 patients. In 2013, there were 18 PC centres in the Turkey [43]. By 2017, the number of PC services increased to 227 PC Units, 947 home care teams and trained 21,696 family physicians within the MoH organisation [44]. A study in 2018, presented that there were four paediatric PC hospital services in Turkey [136]. As of February 2020, there were 415 PC centres, with 5,577 bed capacity and 6,011 PC workers in 81 Provinces. Among these, there were 10 paediatric PC services with 119 bed capacity. More than 290,000 patients got support from these services [137] (Figure 4).

The increase in PC centres also resulted in an increased number of research outputs. A study showed that 9% of 214 patients receiving home care health services in Kirikkale city had a diagnosis of cancer [138]. A study reported 45,838 out of 409,337 patients with respiratory problems benefitting from home care services had a diagnosis of lung cancer during 2011–2017 and the number of lung cancer patients increased from 1,346 in 2011 to 9,206 in 2017 [139]. Another study showed that cancer was the fourth most common diagnosis among patients receiving home care services. During the period of 2011 and 2017, total number of home care visits increased from 3,440,144 to 10,917,965; the number of home care teams increased from 593 to 662. Among home care visits, number of cancer patients increased from 7,278 to 74,261 [140].

With the increase in the number of services, PC units around the country started to report their experiences [141–145]. Most of the studies focused on symptom management [146–171]. Different aspects of supportive care were investigated in many studies including quality of life [172–181], care dependency [182], nutritional aspects [160, 183, 184]; complementary approaches like acupress [185], reflexology [186], lymphedema management [187], bio-resonance [188] and music therapy [189]. A study from a PC centre showed 87.8% of 327 patients had cancer during 2015 and 2017. The most common reason for hospitalisation was oral intake impairment in 34.6%, pain control in 25.5% and both in 16.2% [190]. Ozcelik *et al* [191] prepared a PC guideline in 2014. Erkal *et al* [192] discussed the cost, management, satisfaction and other issues in PC services.

A few studies investigating emergency department admissions showed that 20%–60% of patients had a diagnosis of cancer [193–195]. A mortality analysis of 373 stage 4 cancer cases showed that 23.9% were given chemotherapy during a month before their death [196]. A study investigated the last 2 weeks of 422 terminal cancer patients in hospital; the invasive pain management was used in 25%, terminal sedation in 12%, chemotherapy in 9%, central catheters in 38%, transfusion in 43% and MR imaging in 13% [197]. Another study in cancer patients showed that 42% of patients were given palliative chemotherapy in the last 3 months of life [198]. There are similar studies that focused on the interventions and investigations during the last weeks of life [199–201]. These studies highlight unnecessary procedures at the terminal stage and emphasise the need for PC among cancer patients.

Symptom control was not at the optimal level. A multicentre study in 1,245 lung cancer patients found that pain was controlled in only 21.7% cases, while dyspnoea in only 12.4% of cases [202].

Poor symptom control also affects home based care. A study among caregivers of terminal cancer care presented that most participants expressed that they would like to look after their patients at home, however they preferred hospital care at the end [203]. A study shows that the health literacy level of care givers has a significant effect on bedsore occurrence and survival [204]. A positive correlation between ‘the time from diagnosis to palliative care application’ and the quality of life was found in a cohort study [205].

An analysis on the nosocomial infections in PC unit showed that the average cost of antibiotics was 1,252.79 ± 1,616.50 Turkish Lira (TRY) [206]. A cost comparison was made among cancer patients treated at comprehensive PC; hospital in-patient PC and home health care services, and it was found that the mean total indirect costs were $164.10, $778.43, $344.62, respectively. The mean total direct costs were $2,384.57 and $4,775.68 in comprehensive palliative care services (CPCC) and hospital inpatient palliative care services (HIS), respectively [207]. A cost analysis in a medical intensive care unit where 77% of the patients had terminal cancer found that the median cost was 2,841 TRY, and the total cost was 581,353.2 TRY [208]. A cost analysis in Denizli State Hospital PC Centre revealed that total cost of the PC centre was 1.034.235,26 TRY [209]. In another study, the direct cost per patient per day in a PC centre was found as 391 TRY [210].

A survey showed that the patient satisfaction was higher in oncology centres having PC units [211]. A study showed that PC centre was less effective in reducing symptom levels in cancer patients compared with patients in general care at the public hospital, but provided greater patient satisfaction [212].

### Public awareness, psychosocial support and end of life ethics

A low level of public awareness was identified in many studies. It was found that 60%–87% of cancer patients and their caregivers had no prior knowledge about PC [203, 210, 213, 214]. Home care awareness was found as 57% [119]. Another study presented that patient preference for home care increased from 12% at admission to 47% at discharge [215]. Quality of life for care givers was also investigated in a study, and it was found that 53% of the study subjects did not meet routine responsibilities [216].

Do not resuscitate (DNR) is an important element of PC and end of life. However, there is no DNR Law in Turkey. Many researchers stressed the need for legal arrangements for end of life care and DNR policies [20, 217–220].

Along with progress on PC at national level, many studies were conducted on the subjects of psychosocial issues [221–234], patient and professional satisfaction [235–237], burden of the caregivers [238–247] and end of life [248–251]. A survey among doctors and nurses in Middle Eastern Countries including Turkey showed that 44% of participants provided spiritual care less often than they think they should [252]. A study among PC nurses found that they perceive death as a natural and inevitable process and as their experience increases, they become desensitised [253]. A study showed that 55.7% of 70 nursing students had never heard of spiritual care [254]. Another study investigating the cultural mourning ritual, the ‘First Feast’, found this tradi tion helped to ease the grief response of relatives and might be a useful auxiliary method for PC teams to help grieving families [255]. There are other studies that focus on the psychosocial and spiritual issues on caregivers [256–258].

A study also found that 80% of the caregivers had inadequate health literacy regarding general health [259]. Barriers for PC development were discussed in some studies [20, 24, 34]. The major barriers reported by Turkish MoH, 2nd Turkish Medical General Assembly Clinical Oncology Study Group were lack of public and professional awareness, failure of PC planning and disconnection from anticancer treatment, hurdles in the accessibility of opioid analgesics, financial issues and the lack of trained PC providers [260].

## Discussion

The population is ageing in Turkey, the proportion of people over 65 years of age comprised 9.5% of the population in 2020 [8]. The need for PC in Turkey has increased in parallel to the burden of NCDs and cancer. The estimated annual deaths due to NCDs in Turkey was 407,300, it makes 89% of total deaths in 2016 [261]. Cancer is listed as the second most common cause of mortality which accounts for 18.4% of all deaths in Turkey during 2019 [262]. There were very limited services mainly provided during the routine care in tertiary care hospitals before 2000. Indeed, the situation was not much different at a global level – it was reported that globally over 56.8 million people are in need of PC each year. Most of them are living in developing countries [5]. The EAPC published that the number of PC beds per million inhabitants was 45–75 in advanced European countries, the rest had few beds [133]. Both professionals (mainly oncologists and anaesthesiologists) and government have contributed on the progress of PC after 2010 significantly in Turkey. The partnership between oncology societies and cancer control department at MoH became a successful model for the planning and implementation of PC services in Turkey. MoH actions in PC boosted the involvement of other stakeholders, regional/international organisation. The PC activity was scaled up to group 3b in 2011. After the implementation of Palliaturk project in 2011, various PC related legal regulations were enacted [6, 12, 33–35]. During the same period, the demand for PC due to changing demographic patterns (ageing, decrease in household size) and burden of cancer/NCDs was also increasing.

Many studies showed that awareness and knowledge about PC among medical professionals was very limited. PC is still not a recognised speciality in Turkey. Only a few universities include PC education in the under and postgraduate curriculum. National and international meetings were the main source of PC training. There is still a large variation in palliative medicine (PM); education even in Europe. In the WHO Europe region, PM courses were included in all medical schools of 13 out of 43 countries, PM was not taught within medical curricula in 14 countries [263]. Major oncology associations like the American Society of Clinical Oncology (ASCO) & ESMO are also investing in the integration of PC services in oncology [264]. During the last 10 years, PC medicine is gaining more investment from all stakeholders. Sedhom *et al* [265] commented that PC is still not integrated into cancer care with such a priority focus of oncology training on treatment and research. There is still a strong need for an advanced education and structured human resource policies. An early literature review in Turkey showed the limited PC publications before 2005 [266]. The number of scientific publications and presentations started to increase after 2015; however, most of the studies were either surveys or descriptive studies. More focus on evidence-based research in PC is still needed. A bibliometric analysis on PC during 2000–2016 showed that the publications increased after 2006, the USA and UK were pioneering the scientific work [267].

The use of opioids is being recommended for the control of the pain and improving quality of life [5, 229], and it is used as an indicator for the PC service availability [268]. There were strong critics to Turkey for underuse of opioids for pain management, although Turkey was one of the leading opioid producing countries [34, 43, 121]. The situation improved after the investments on PC starting from 2010, however, it is still much lower than the global average [34, 43]. Consumption in defined daily doses for statistical purposes (S-DDD) per million inhabitants per day is within the scale of 201–1,000 during 2014–16 which was less than North America and Europe but higher than most Asian and African countries [269]. Based on our literature review, we can classify the barriers to opioid use into three groups: first is the lack of awareness and opiophobia among the health care professionals and public; second the complexity in prescription of the opioids due to legal procedures and thirdly the lack of organised PC structure until 2010. There is still a need for investments in the infrastructure, training and human resource management on PC under the concept of ‘health system strengthening’.

Before the implementation of the Palliaturk project, few hospitals and universities started to invest on PC services, there were no specialised PC centres in those years [20, 21, 23, 26, 43, 133]. The government Palliaturk project, the PC Directive and the Home Care Regulations contributed to the progress made during recent years [12, 20, 39, 41]. Currently, there is a PC section within MoH at top management level and MoH was able to invest in the dissemination of PC capacity in Turkey [137]. Our findings show that integration for PC services with oncology services is an area for further research and investment. There are a number of cultural barriers and opportunities for the utilisation of PC centres in Turkey. Talking about death is a cultural taboo. Doctors, patients and care givers are reluctant to talk about end of life care. This results in delayed referral to PC centres by professionals. Moreover, the concept of PC centres is not clear in the minds of the public. Therefore, referral to PC centres was sometimes perceived as an abandonment of oncological care. Due to this, patients and caregivers want to be under the care of the primary oncology team instead of PC centres. The integration of PC care into oncology practice will help to overcome these cultural barriers. Traditionally, terminally ill patients were cared for in their homes by family members. However, with social and economic changes, care givers now more commonly prefer their patients to be cared for in the hospital. This could be an opportunity for the utilisation of PC centres more widely. ASCO as a leading professional cancer organisation also strongly advocates the integration of PC into oncology practice to disseminate and implement it more effectively [270].

Currently, there is no DNR Law in Turkey [20, 217]. Moreover, the Turkish society is not ready to implement DNR policies due to several reasons such as health literacy, taboo of discussing death and lack of legal framework. Therefore, DNR is not a priority issue either for the public or government. End of life care and DNR policies must be brought in to the discussion at the public, professional and government level. It is also a time for Turkey to invest in hospice care. The Turkish Society of Internal Medicine also declared that they support Choosing Wisely®, a health initiative campaign with recommendations to advance a national dialogue on avoiding unnecessary medical tests, treatments and procedures and ‘Do not delay the palliative care’ is among the recommendations by this campaign [271]. A recent article by Currow *et al* [272] discusses the need for transition of hospice care and integration of cancer services with hospices care. It seems the hospice concept will be evolved through the changes in the cancer care in future years.

Now, monitoring the global situation of PC is highly essential. The burden of chronic diseases was in the agenda of UN General Assembly in 2011 and the international organisations/NGO’s started to speak more about the need for PC. Due to the increased burden of NCDs including cancers, Turkey must scale up its PC services. One important issue is the effect of the COVID-19 pandemic on cancer care including PC [273, 274]. An NCD pandemic is a high possibility in the post-COVID-19 period and a greater need of PC is expected.

The International Association for Hospice and Palliative Care re-defined PC as ‘Palliative care is the active holistic care of individuals across all ages with serious health-related suffering due to severe illness and especially of those near the end of life. It aims to improve the quality of life of patients, their families and their caregivers’ [275].

## Conclusion

In conclusion, this review presents evidence of the significant progress made in Turkey during the last 20 years but also presents the opportunities for further improvement. Bridging the gaps in human resources and training, including PC care as a priority area in the national health agenda, the commitment of all stakeholders, and investing in public and professional awareness should be the focus of the next steps and also shaping the integration of PC in cancer care in Turkey. The inequity in PC must be a priority action for decision makers not only for Turkey but also globally. The stakeholders and decision makers should not neglect the need for PC improvement under the current pressure of the COVID-19 pandemic on health and the economy at a national and global level.

## Conflicts of interest

The authors declare that there are no conflicts of interest.

## Authors’ contributions

TK, FA, MC and MK contributed to the (I) Conception and design, (II) Administrative support, (III) Provision of study materials, (IV) Collection and assembly of data, (V) Data analysis and interpretation, (VI) Manuscript writing: All authors and (VII) Final approval of manuscript.

BA, MŞ and SY contributed to the (I) Conception and design, (III) Provision of study materials, (IV) Collection and assembly of data, (V) Data analysis and interpretation, (VI) Manuscript writing and (VII) Final approval of manuscript.

RS and RH contributed to the (I) Conception and design, (V) Data analysis and interpretation, (VI) Manuscript writing and (VII) Final approval of manuscript.

## Funding

This work was supported by The UK Research and Innovation Global Challenges Research Fund (GCRF) Research for Health in Conflict in the Middle East and North Africa (R4HC-MENA) project; developing capability, partnerships and research in the Middle East and North Africa [ES/P010962/1].

## Supplementary appendix

The supplementary tables are also available online at https://doi.org/10.6084/m9.figshare.17057009.v1.

**Supplementary Table 1.** Details of the included studies.

Author; year (Ref.#)	Language	Journal paper/conference presentation/poster	Research design	Aim/obj. of study	Key findings/observations
Silbermann *et al* [22]	English	Journal paper	Review	This article reviewed palliative cancer care in Middle Eastern countries.	Before 2010, palliative care (PC) was confined to only pain clinics. Turkey was at number 50th, for the consumption of opioids as analgesics among all countries. After The Pallia-Turk project, 100,000 patients have been treated during the first 8 months of the year 2011 and the number of home visits for PC was 230,000.
Al-Shahri [276]	English	Journal paper	Review	This article reviews the status of PC in seven Islamic countries.	Ongoing activates in Turkey for the implementation of PC programmes and education. In 1993, the Turkish Society of Algology became a regular chapter of the International Association for the Study of Pain.
Bagcivan *et al* [98]	English	Journal paper	Tool validation	To evaluate validity and reliability of the Barriers Questionnaire II (BQ-II) in Turkish cancer patients.	Barriers Questionnaire II, BQ-II is a valid and reliable scale to identify the patient-related barriers for cancer pain control in Turkey.
Bingley and Clark [19]	English	Journal paper	Review	In this study PC, development and services were reviewed in the six countries of the Middle East Cancer Consortium.	PC in Turkey was at the very early stages of ‘capacity building’. Specialist PC services and funding did not exist. Terminally ill patients have access to symptoms management. Only a few consultants in oncology or pain units were trained to provide PC. Along with legislative and practical obstacles, the opioid consumption was relatively low.
Bulbul *et al* [202]	English	Journal paper	Cross-sectional	To investigate the symptoms among lung cancer cases in Turkey and approaches to alleviate these symptoms.	The common symptom among 1,245 lung cancer cases was tiredness (82.1%), followed by dyspnoea (69.3%), loss of appetite (65.7%), pain (65.4%), drowsiness (60.8%), anxiety (57.7%) and depression (51.1%). The symptoms were more severe for stages III and IV patients. Symptom control was insufficient even after treatment. The pain was controlled in only 21.7% of cases, while dyspnoea in only 12.4% of cases and appetite stimulant was useful in only 18.9% of cases.
Çınkır and Kahraman [190]	English	Journal paper	Retrospective cross-sectional	To evaluate the data of patients treated and followed-up in PC service of a training and research hospital.	Of the 327 patients, 58.1% were male. The median age was 63 years. The mean hospital stay was 8 days. About 87.8% of the patients had cancer and cancer-related complications. The most common cancer was the stomach (32.7%) followed by the oesophagus (11.3%). The most common reason for admission was oral intake disorder (34.6%), pain (24.5%) and pneumonia (15.0%). In a follow-up study, 77 patients (23.5%) died within 2 years.
Cleary *et al* [120]	English	Journal paper	Review	This study was aimed at identifying opioid availability and accessibility in the Middle East.	There are no restrictions on the eligibility of a patient for opioid analgesics in Turkey. Physicians can prescribe opioids for up to 10 days. The Statistical purposes Daily Dose for per Day per million people (S-DDD) was raised from 120 mg during 1997–1999 to 580 mg in the year 2007–09.
Dincer *et al* [115]	English	Journal paper	Cross-sectional	This study was planned to determine the attitudes of nurses in PC centres towards the patient safety culture.	Out of 487 PC nurses, 76.5% admitted that they had received patient safety training. Perception of the patient safety culture using the Patient Safety Culture Scale was found positive. 94.2% admitted an increased risk of errors because of excessive workload. 82.1% thought that paucity of logistical and resources affected the patient safety culture.
Elcigil [52]	English	Journal paper	Review	This review provides an oncology nurse’s perspective regarding the status of PC.	A significant proportion of the Turkey population does not have access to PC and most terminally ill cancer patients were treated by pain specialists and medical oncologists. Barriers to the development of PC nursing were; Lack of certified PC education and training programmes, limited research, lack of public awareness, shortage of nursing staff and limited knowledge about opioid analgesics use.
Emuk and Naz [58]	English	Journal paper	Review	This article briefly described the Pallia-Turk project and highlighted the situation of PC in Turkey.	The aim of the Pallia-Turk project was 1) to increase morphine availability, 2) legislations for morphine prescription, 3) training of medical staff and 4) implementation of a community-based PC programme. The project consists of primary, secondary and tertiary level PC centres. PC was the weakest part of the National Cancer Control Programme. The project needs to clarify education, training medical ethics and standardise tools to evaluate psychosocial needs.
Ertaş *et al* [277]	English	Journal paper	Clinical trial	To evaluate the effectiveness of subcutaneously implanted epidural ports in the management of advanced-stage gynaecologic cancer pain.	Morphine administration via subcutaneously implanted epidural ports provides excellent pain relief in gynaecologic cancer without side effects and increased patient quality of life.
Eyigor [73]	English	Journal paper	Cross-sectional	To evaluate the knowledge of medical students on PC and their views on PC in clinical practice.	Among 175 fifth-year, medical students most gave correct answers about PC philosophy. Concerning symptom control, the answers were mostly incorrect. 64.6% stated that the education on PC was insufficient and 90.3% did not receive training on end-of-life communication skills.
Goksu *et al* [196]	English	Journal paper	Retrospective/cross-sectional	To evaluate the aggressive use of chemotherapy among cancer patients in Turkey near the end of life.	A total of 373 stage IV solid tumours patients died between years 2010 and 2011. Of these, 23.9% underwent chemotherapy a month before their death. The likelihood of chemotherapy was influenced by patients’ age, more recent diagnosis and performance status. No association was found between gender and tumour type with chemotherapy.
Hacikamiloglu *et al* [43]	English	Journal paper	Review	This review article described the Community Palliative Care programme in Turkey.	PC training programmes were organised in collaboration with the Turkish MoH and the Middle East Cancer Consortium (MECC) between 2004 and 2014 and a total of 434 people participated. Among the participants, 27.1% were nurses and 26.9% oncologists. The MoH launched a national home-based PC programme with 407 home care teams in 2010. By the year 2014, there were 834 home care teams providing services to 416,175 patients. In addition to that, there were 18 PC centres in the country.
Hajjar *et al* [18]	English	Journal paper	Review	This review article focuses on the prevalence of the ageing population in the Middle East including Turkey and its implications on cancer incidence and care.	In the year 2000, about 13.1% of all deaths in Turkey were due to cancer and it is projected to rise 16.2% in 2030. In 2006, direct cost for cancer treatment constituted 11% of overall healthcare expenditure. Regarding terminal stage cancer patients ‘home care’ is generally provided by relatives in Turkish society.
Isıkhan [47]	English	Journal paper	Review	This review article highlights the current level and future status of social work in PC services in Turkey.	The effort for PC for cancer patients was started with the initiative of TMoH Fight Against Cancer in the year 2003. The psychosocial subcommittee was formulated and a training programme for social support was discussed. By the year 2015, there were 31 certified PC centres with 389 beds and 50 social workers providing support in oncology units of hospitals. Difficulties experienced by National Cancer Advisory Board were; excess workload, difficulty in identifying PC needs, reluctance to discuss death, ineffective communication skills and lack of training of health personnel.
Iyigun *et al* [110]	English	Journal paper	Tool validation	To examine the psychometric properties of the Turkish version of the Cancer Behavior Inventory-Brief Version (CBI-B).	The Turkish version of the Cancer Behavior Inventory-Brief Version was a valid and reliable inventory for the psychometric evaluation of Turkish cancer patients coping with cancer.
Kahveci and Gokcinar [213]	English	Journal paper	Cross-sectional	To determine the level of knowledge of PC among the family members of patients requiring PC.	Among 150 family members of patients receiving PC, 68.0% had no prior knowledge of PC. In all, 50.7% of the participants thought that PC could prolong life, and 32.7% thought that PC was designed to be curative.
Karaoren *et al* [193]	English	Journal paper	Retrospective/cross-sectional	To determine the need for a ‘hospice unit’ in Turkey.	One year (2010–2011) record of 287 patients from the emergency department of a university hospital found that approximately half of all patients had a diagnosis of cancer, and 47% of those had grade IV disease. 63.8% of patients for whom intensive care monitoring was recommended were over the age of 60 years, 20% had advanced-stage cancer and the predicted mortality rate was nearly 60%.
Kinay [46]	English	Journal paper	Review	This article describes the establishment of a PC unit in a university hospital.	Describes the efforts for establishment of a multidisciplinary PC unit in the oncology department of a university hospital and activities for the initiation of MS and Ph.D. programmes in Palliative and Supportive at the university.
Komurcu [16]	English	Journal paper	Review	This review outlines the history of PC development in Turkey.	The concept of PC in Turkey was started after the establishment of the Association of Pain management in 1998. Subgroup for Supportive Care in Cancer was formulated within the Turkish Oncology Group and efforts for the development of PC programme in turkey were initiated after 2000.
Lynch *et al* [33]	English	Journal paper	Review	This descriptive study aimed to categorise the countries according to PC development and to present changes in PC over time.	All countries around the globe were classified into six groups based on PC service provision. Turkey was placed in Group 3b (generalised PC provision) along with Albania, Argentina, Belarus, Bosnia and Herzegovina, Cote D’ivoire, Croatia, Cyprus, Czech Republic, Georgia, India, Jordan, Lithuania, Malta, Nepal, Portugal and Swaziland. It was cited that there were 14 PC services available in the country and the Ratio of Palliative Care Services to Population was 1: 5344000.
Mojen *et al* [135]	English	Journal paper	Review	In this study paediatric PC system in Egypt, Lebanon, Jordan, Turkey and Iran has been summarised.	The first PC unit for children established in 2011, at Dokuz Eylül hospital in Izmir. In- and out-patients services were provided by the multidisciplinary team of doctors, nurses, psychologists and social workers. Training workshops, to create a model of PC, were held and a total of 434 people in Turkey had been trained in PC by 2014.
Mutafoglu and Group DEUPCS [134]	English	Journal paper	Review	This article describes establishment of PC unit in a university Hospital.	Institutional efforts for establishment of PC unit in a university hospital in Turkey. It also describes how a multidisciplinary PC team provides in and outpatient services.
Özmen *et al* [260]	English	Journal paper	Review	This article presents the summary of The 2nd Turkish General Medical Assembly Clinical Oncology Study Group meeting held in Istanbul in October 2015.	It was emphasised that PC should be integrated as a component of cancer treatment, through a multidisciplinary approach, PC training should include physicians, nurses and healthcare staff, the care should be based on a patient’s need and wishes and the concepts of national PC should be made in line with the World Health Organization criteria; moreover, necessary legal arrangements should be provided for the right for DNR and regulations on terminal sedation decisions.
Ozgul *et al* [12]	English	Journal paper	Review	This article reviews opioid availability, accessibility and regulatory barriers for cancer patients in Turkey.	There were 72 pain control units and only 10 PC centres across the whole country. The morphine consumption rate per capita was low, and the accessibility, as well as the availability of morphine products, is limited in the country. Family physicians will have the right to prescribe opioids.
Ozgul *et al* [28]	English	Journal paper	Review	This manuscript summarises the situation before the Pallia-Turk Project and updates of 2 years period after of that.	The two main objectives of the Pallia-Turk Project were the availability of opioids and the implementation of a community-based PC model. The availability of different types of morphine in the markets has been increasing through new legislation. The medical staff will be trained against opiophobia. A telephone number had been established for patients in need of home care. Out of 190 home visits, most had neurological diseases and only 4% had cancer.
Ozcelik *et al* [107]	English	Journal paper	Tool validation	This study aimed to perform a psychometric evaluation of the (EORTC QLQ–C15–PAL).	The Turkish version of the European Organization for Research and Treatment of Cancer Quality of Life Questionnaire–Core 15–PAL is a valid and reliable tool to determine the quality of life of advanced cancer patients who are undergoing palliative treatment in Turkey.
Ozcelik *et al* [106]	English	Journal paper	Review	To determine the level of satisfaction of 145 family members of patients with advanced-stage cancer receiving PC.	The average total family satisfaction score was 76.87 ± 1.14. A significant relationship was found between the family member’s level of satisfaction and possession of sufficient information about the patient.
Polat and Karatas [80]	English	Journal paper	Cross-sectional	This study was aimed to determine nursing students’ knowledge about PC.	Out of 658 nursing students, only 5.3% correctly answered all items about PC, while 26.3% gave correct answers in line with the PC theoretical framework and 5.3% gave correct responses for symptom management.
Sahin *et al* [101]	English	Journal paper	Cross-sectional	Relationships between demographic variables and hopelessness and depression among terminally ill cancer patients.	The hopelessness score of 216 terminally ill cancer patients was significantly higher for cancer patients who were; female, illiterate, married and living in rural areas. Hopelessness and depression were significantly higher for those having; longer duration of disease, receiving radiotherapy and advanced metastatic disease. The significantly negative association between hopelessness, depression and perceived social support from family was evident.
Seven *et al* [108]	English	Journal paper	Cross-sectional	To identify gynaecologic cancer patients’ PC needs.	Out of 134 gynaecologic cancer patients with PC needs, 69.4% had ovarian cancer and of all 52.2% had stage-3-4 cancer. 33.5% expressed as they were a burden for their families and about 28.4% feel lonely at some level. The most prevalent unmet needs were tiredness (60.5%), feel depression (47.4%) and lack of appetite (38.5%), pain (38.1%) and nausea (26.3%).
Silbermann [121]	English	Journal paper	Review	This review article determines and compares the medical use of opioids consumption in Middle Eastern Populations.	Between 2004 and 2007, the consumption of morphine has been fluctuating at doses of about 0.1 mg/capita in Turkey.
Silbermann *et al* [34]	English	Journal paper	Survey	To determine knowledge, beliefs, barriers and resources regarding PC services in Middle Eastern countries.	Cancer patients were informed by treating physicians about different care options including PC. The opioid consumption in Turkey during 2011 was 12.220 milligrams per capita and it was five times lesser than the global mean value.
Süren *et al* [95]	English	Journal paper	Cross-sectional	This descriptive study was aimed to investigate the symptoms and needs of terminal cancer patients.	Of 107 patients, 43% had gastrointestinal and 25.3% had genitourinary cancer. The common symptoms were fatigue (98.1%), pain (92.5%), insomnia (92.5%), loss of appetite (76.6%), constipation (71%), dyspnoea (63.6%), nausea (60.7%), cough (57.9%) and vomiting (48.6%). Formal PC is limited in northern Turkey and most patients were referred at the late stages of the disease and with the severity of the symptom.
Turgay and Kav [76]	English	Journal paper	Cross-sectional	To explore health professional’s view of PC.	Among 369 healthcare professionals, most of the respondents admitted lack of continuing PC education programme and more than half affirmed that they had never received education in palliative care. However, the majority of the respondent had a clear meaning of PC and make the correct option.
Oğuz *et al* [217]	English	Journal paper	Review	This article review end-of-life care in Turkey.	With the population ageing, the inadequacy of end-of-life healthcare is a critical issue for Turkey. The healthcare provider must learn to communicate the risks and benefits of medical procedures and alternatives treatment with terminally ill patients as well as their relatives. Legislative procedure for DNR and public discussion about end-of-life care are needed.
Yildirim and Tanriverdi [195]	English	Journal paper	Retrospective/cross-sectional	This retrospective study focused on how often and why patients with cancer visited the emergency department (ED) of a university hospital before death.	The medical record of 107 deceased cancer cases showed 60% of cases had at least one visit to ED within 1 month before death. Of these, 38% were discharged, 9% died in the ED while 53% were hospitalised. The most common site of the primary tumour was the lung (38%), the three most common presenting symptoms were dyspnoea/shortness of breath, pain and deterioration in general health status. The most common reason for hospitalisation was ‘social support’.
Zeybek Unsal and Büken [218]	English	Journal paper	Review	To examine existing legislative regulations on PC in Turkey and compare with Europe.	Patients’ Rights Implementation Directive has provisions for patients to refuse treatment or withdrawing treatment. Article 13 of the Regulation forbids euthanasia. The law distinguishes between assisted suicide and withholding or withdrawing life continuing treatments. The ‘Directive on Implementation Fundamentals and Procedures of Palliative Care Services’ was a force through 07/07/2015 and states that in PC, patients will be informed about patient rights, obligations and details of care service. The patient also has the right to refuse to have PC.
Ozcelik *et al* [21]	English	Journal paper	Review	To describe PC activities in the field of oncology in Turkey.	PC services were not provided at the specialist level in Turkey. Barriers to the provision of PC were; lack of trained personnel, social security coverage for home-based PC cost, legal issues and opioid phobia. Turkey ranks 44th among 51 countries of the WHO European region for per-capita opioid consumption. The right of dying patients to issue advanced directives has not been established by law in turkey and the DNR order is not legal.
Ben-Arye *et al* [88]	English	Journal Paper	Cross-Sectional	Integration of complementary medicine in supportive cancer care in the Middle East.	The use of complementary and traditional medicine (CTM) among patients with cancer in the Middle East ranges from 35% in Iran to nearly 100% in Jordan. Use of CTM is 57% in Turkey. Only limited research has been published on the integration of CTM in cancer care across the Middle East.
Uslu-Sahan *et al* [79]	English	Journal paper	Cross-sectional	To assess knowledge, practice and opinions about barriers toward PC among nurses working in gynaecologic oncology units.	Among 65 nurses working in gynaecologic oncology units, 52.3% did not receive any knowledge regarding PC. The training received was communication skills (96.8%), pain management (96.8%) and symptom control (96.8%). About 73.8% did not talk about death with patients and their families. One of the most common barriers faced by nurses was opioid phobia experienced by patients (66.2%) and healthcare professionals (41.5%).
Erdine *et al* [15]	English	Journal paper	Review	A concise overview of early days of PC in Turkey.	Pain & PC have been the important target of the Turkish Society of Algology. Palliative care Campaigns of the WHO were organised by the Turkish Society of Algology. WHO Cancer Pain management booklets were translated into the Turkish language.
Alkan *et al* [109]	English	Journal paper	Cross-sectional	To evaluate the predictors of Persistent postmastectomy pain syndrome (PMPS) and posttraumatic stress disorder (PTSD) in breast cancer survivors.	Among 614 breast cancer survivors, the incidence of PMPS was 45.1%. PTSD was documented in 75% of cases. Low income, presence of PTSD and <46 months after surgery were associated with increased risk of PMPS.
Büken [278]	English	Journal paper	Review	In this paper, the concepts and application of medical ethics specific to end-of-life PC were discussed.	The physicians encounter many ethical dilemmas coming at the end-of-life stage of patients with cancer. Dealing with these dilemmas, the decisions of the interested parties (physician, medical personnel, patient, patient’s family, health institution) about the actuation of the clinical and ethical decision-making processes are affected by the society’s moral, social, cultural and legal structure as well as the health policy and health economies of the state.
Sert and Guven *et al* [219]	English	Journal paper	Review	This paper examines the ethico-legal problems regarding the right to refuse treatment in Turkey’s.	Article 17, paragraph 2 of the 1982 Constitution of the Republic of Turkey provides a legal framework governing the limits of the right to refuse treatment in Turkey. However, interpretation of the scope of this framework is complex and it is needed to be clarified and amended under international conventions and fundamental human rights.
Tanrıverdi *et al* [102]	English	Journal paper	Cross-sectional	To evaluate the caregiver’s burden and prevalence of depression among caregivers of cancer patients receiving chemotherapy.	Depression was found among 64% of the caregivers. The presence of depression was associated with young age, being female, high education, low economic status, financial loss during treatment, patient’s lack of knowledge about his/her diagnosis, metastatic disease and short survival time. It was found that among 968 participants 36% had suffered financial loss, 56% had incurred diminished social activities, 42% confirmed a lack of self-care and 18% had lost their jobs. The patient’s lack of knowledge of the diagnosis was the independent risk factor caregivers burden.
Tanrıverdi *et al* [194]	English	Journal paper	Retrospective/cross-sectional	Identify the characteristics of cancer patients admitted to the emergency department in Southwest Turkey.	A total of 304 emergency department visits by 102 cancer patients between August 2011 and September 2013. The majority of patients were male (65%) and over 65 years of age (52%). About 30% had lung cancer, among all 32% presented with dyspnoea, 53 had metastasis. The Eastern Cooperative Oncology Group-ECOG performance status was poor (score 3 to 4) among 68% of patients.
Walker *et al* [279]	English	Journal paper	Review	This paper summarises the status of PC in the United States, Turkey, and Malawi. It also describes collaborative projects in cancer and PC in these countries.	The University of Alabama at Birmingham School of Nursing (UABSON), through a Pan American Health Organization/World Health Organization Nursing Collaborating Center, has initiated collaborative projects in cancer and PC between the USA, Turkey and Malawi, to strengthen initiatives that can ultimately transform the practice. The UABSON has collaborated with Turkish nurses and researchers to develop a PC network and research.
Yüceege *et al* [105]	English	Journal paper	Tool validation	To identify the clinical utility of Memorial Symptom Assessment-Short Form and Condensed Memorial Symptom Assessment Scale in Turkish lung cancer patients.	Memorial Symptom Assessment-Short Form and Condensed Memorial Symptom Assessment Scale can be used in lung cancer patients The Cronbach’s alpha coefficients were 0.861 (1.785–0.915) for MSAS-SF and 0.728 (0.566–0.835) for CMSAS. Scores from both assessments are significantly higher in patients with metastatic disease, and additionally, all of the MSAS-SF subscales were significantly higher in metastatic patients.
Kucukoner *et al* [89]	English	Journal paper	Cross-sectional	To determine the frequency, type and associated factors of complementary and alternative medicine (CAM) by cancer patients.	It was found that at least one CAM method was used by 62% (*n* = 201) of the patients. 82.5% of patients treated with CAM were using at least one herbal species. Among CAM users, 40.9% were using herbal mixtures and 39.8% were using *Urtica dioica*. CAM was preferred more frequently by the patients, age 40–60 with metastatic stage, receiving palliative treatment, chemotherapy.
Soylu *et al* [93]	English	Journal paper	Cross-sectional	To explore advanced breast cancer patients’ knowledge of treatment intent and expectation of illness course and to evaluate their relationship with optimism, hope and quality of life (QoL).	Out of 55 advanced breast cancer patients, treated in the ambulatory clinic of the University Hospital, 32 (58.2%) patients had an inaccurate perception of treatment intent, believing the aim of treatment was the cure. Regarding the expectation of disease course, only 21 (38.2%) had a realistic expectation that their disease may remain stable or may progress over a year. Also, the awareness of disease progression and perception of goals of treatment was significantly related to hope and optimism scores but not with QoL.
Kafadar *et al* [64]	English	Journal paper	Cross-sectional	To evaluate the managerial perspectives and opinions of the hospital managers and clinical directors about specialised PC centres.	Interviews were carried out with 70 hospital managers and clinical directors in two oncology hospitals in Istanbul. The need for establishment of PC unit in the hospital was suggested by most (87%) of participants. Participants also revealed institutional social and educational factors as potential barriers in providing PC and integrating PC into health systems. To overcome the barriers for integration of PC into health systems, providing education for health professionals and patient relatives, raising awareness in society, financial arrangements and providing infrastructure were suggested.
Oktay *et al* [74]	English	Journal paper	Cross-sectional	The perspectives of 4,224 medical students on cancer, its treatment, PC and the role of oncologists.	The result showed that oncology education among phase III to VI students was useful for the students’ understanding of cancer, cancer screening and prevention. However, oncology education to phase III to VI students failed to conceptualise the true meaning of palliative/supportive care and student thought that it was the oncologist’s responsibility to treat symptoms such as pain, nutrition and shortness of breath in cancer patients.
Müller-Schwefe *et al* [280]	English	Journal paper	Cross-sectional	Multinational survey to understand the perspective of patients with chronic pain treated with opioids on quality of treatment, compliance and communication between patients and physicians.	Of the patients surveyed, 61% received strong opioids. Nearly 65% of the patients were on a twice-daily or more dosing schedule; however, 61.5% considered once-daily dosing to be the most convenient schedule. Patients’ responses indicated that different dosing schedules significantly influenced the occurrence of end-of-dose pain, feeling limited by the remaining level of pain, problems in falling asleep and episodes of waking up at night or early in the morning. A total of 556 physicians also participated in the survey. A majority of the physicians reported that they were not surprised by the patients’ responses regarding medication and wanted to change some aspects of pain therapy such as changing the medication, co-medication and non-medical therapy.
Bar-Sela *et al* [252]	English	Journal paper	Cross-sectional	To examine the extent of spiritual care provided by staff and also staff attitudes toward spiritual care, including perceived barriers.	The survey was conducted among 770 physicians, and nurses caring for patients with advanced cancer from 14 Middle East countries. The 168 respondents were from Turkey. Nearly one-half of the participants admitted that they would like to provide spiritual care more often than they do. The most commonly perceived barrier to care provision was insufficient training and 77% of respondents lacked such training. The single strongest predictor of not providing spiritual care was not personally relating to spirituality.
Oğuz *et al* [26]	English	Journal paper	Review	This paper describes the Palliative Care Unit in an Oncology Hospital in Ankara, Turkey.	Ankara Oncology Education and Research Hospital was founded in 1956. Initiative to PC approach began in 1991. The Palliative Care Unit was implemented in 2007. This was the first registered unit in Turkey. The PC team was consisting of 3 pain specialist physicians, 1 anaesthesiologist, 12 nurses, 1 medical secretary and 2 helpers. Patients are accepted from all universities, government or private hospitals.
Senel *et al* [111]	English	Journal paper	Cross-sectional	To determine the frequency of delirium and identify associated factors at the PC of oncology hospital.	The incidence of delirium among the patients with cancer was 49.8%. Subtypes of delirium included hypoactive 49%, mixed 41% and hyperactive 10%. The regression analysis indicated that use of opioids, anticonvulsants, benzodiazepines, steroids, infection, malnutrition, immobilization, sleep disturbance, constipation, hyperbilirubinaemia, liver/renal failure, hypoxia, electrolyte imbalance, brain cancer/metastases were associated with delirium.
Özalp *et al* [100]	English	Journal paper	Cross-sectional	To determine the symptom clusters in 170 inpatients at the palliative care clinic of Oncology Hospital.	The most experienced symptoms by the patients during the week before hospitalisation in palliative care clinic were lack of energy (95.4%), weight loss (91.2%), lack of appetite (89.4%), pain (88.2%), dry mouth (87.6%), feeling sad (87.6%), feeling nervous (82.9%), worrying (81.2%) and feeling irritable (80.6%). The Five symptom clusters identified were; First cluster: pain, feeling nervous, dry mouth, worrying, feeling irritable, weight loss; second cluster: feeling drowsy, numbness/tingling in hands/feet, difficulty in sleeping, dizziness, constipation, I do not look like myself; third cluster: nausea, vomiting; fourth cluster: shortness of breath, difficulty in swallowing, cough, change in the way food tastes and fifth cluster: feeling bloated, problems with urination, diarrhoea, itching, mouth sores, hair loss, swelling of arm or legs, change in the skin.
Ozcelik *et al* [116]	English	Journal paper	Interventional	It was aimed to investigate the improvement in symptoms, quality of life, patient and family satisfaction with care, and direct costs resulting from a PC intervention-based case management model.	The level of decrease in symptom severity in the experimental group patients (PC intervention-based case management model) was more than in the control group (conventional care). The satisfaction level of patients and families in the PC-based case management service was higher than that for conventional service in the control group. No statistical difference was detected between the experimental and control groups regarding health costs and duration of hospitalisation.
Akyar *et al* [281]	English	Journal paper	Review	This review article describes an early PC telehealth delivery models ‘Nurture, Advice, Before Life Ends (ENABLE)’.	ENABLE is a collaborative approach to the care of patients and caregivers, focusing on self‑care management and skills training to empower patients to actively participate and make informed choices about treatment issues. It has demonstrated effectiveness in improving quality of life, symptom relief and survival for patients with cancer in the USA. The authors identified it as a good model to develop PC in Turkey. To improve the effectiveness a culturally sensitive model ENABLE‑TR was developed for supportive care coaching to Turkish caregivers of advanced cancer patients.
Vranken *et al* [122]	English	Journal paper	Review	To identify legal and regulatory barriers to opioid access in European countries (including Turkey).	Across all 11 countries, 778 potential barriers (excluding the language category) were identified. Other than the language category, Turkey had shown more than 40 potential barriers in the following categories; prescribing, dispensing, Usage, trade and distribution, manufacturing, penalties, other (medical activities restricted, violation of privacy, other administrative requirements, limited access to education. The Potential barriers in the legislation and regulations were unclear definition of controlled medicines in its use Turkey.
Dincer *et al* [85]	English	Journal paper	Retrospective/cross-sectional	To evaluate the factors affecting the length of stay (LOS) and discharge of 435 patients from a PC centres (PCC) of a State Hospital.	The mean LOS was 27.2 ± 30.9 days. The mean Glasgow Coma Scale GCS was 11.3 ± 3.3, Karnofsky Performance Scale KPS was 39.6 ± 14.4, mortality was 46.2% in PCC. The most commonly seen diagnoses and comorbidities were hypertension 47.1%, cancer 38.4%, cerebrovascular event 25.7% and diabetes mellitus 22.3%. Percutaneous Endoscopic Gastrostomy (PEG) was applied to 29.4% of patients, tracheostomy to 19.8% and mechanical ventilator support to 5.5% of patients. In the regression analysis, the comorbidities (hypertension, pressure injury), PEG, total parenteral nutrition and infection were found to increase LOS in PCC.
Eyigor and Akdeniz [59]	English	Journal paper	Review	This review article describes the importance of physical activity in PC programmes for cancer patients.	Exercise and rehabilitation approaches in PC programmes for cancer patients affect patients’ symptoms, physical functioning, muscle strength, emotional well-being, psychological symptoms, functional capacities, quality of life, mortality and morbidity positively. Based on scientific data, palliative cancer patients should be recommended to participate in exercise programmes.
Tanriverdi *et al* [81]	English	Journal paper	Cross-sectional	The perspective of non-oncologist physicians regarding their attitudes and beliefs associated with PC for patients with metastatic cancer.	A total of 71% of participants identified all patients with metastatic cancer as being a terminal stage, 62% were unaware of PC techniques, 64% did not know about common supportive care options, 59% were against hospice and 63% had no opinion on resuscitation. It was found that non-oncologist physicians believed that palliative/supportive care is the oncologist’s task.
Çiraci *et al* [104]	English	Journal paper	Cross sectional	To evaluate the feeling of loneliness in terminal cancer patients hospitalised for palliative therapy.	The mean level of loneliness was 53.61 ± 9.29. Analysis of the loneliness level according to patient information revealed that the group of patients aged 29–39, males, literate patients and single/widows experienced higher levels of loneliness.
Bülbül *et al* [96]	English	Journal paper	Cross-sectional	To analyse the impact of cancer symptoms on insomnia and the prevalence of sleep-related problems reported by 1,245 patients with lung cancer in 26 hospitals in Turkey.	The prevalence of insomnia was 44.7%. Among all cases, 48.4% reported difficulty initiating or maintaining sleep, 60.8% reported daytime sleepiness and 82.1% reported fatigue. Female gender, patients with stage 3–4 disease, patients with metastases, with comorbidities and with weight loss > 5 kg had higher rates of insomnia. Also, patients with insomnia had significantly higher rates of pain, nausea, dyspnoea and anxiety.
Can [20]	English	Journal paper	Review	This review describes the scope of PC nursing in Turkey.	PC nursing is not recognised as a nursing speciality in Turkey. There was no curriculum, graduate/postgraduate programmes, for nurses on PC in and the scope of the PC nursing education varies among universities.
Terzioglu *et al* [53]	English	Journal paper	Review	This review provides Turkish Nurses’ Perspectives on Palliative Care to the Cancer Patient in Turkey.	The lack of PC education and training opportunities is the most frequently reported barrier to the development of PC in Turkey. PC education has not been integrated into the curriculum of nursing at both undergraduate and postgraduate levels. Nurses acquire his/her PC knowledge and skills after graduation, from programmes with different formats such as workshops, seminars and courses.
Gurhan [24]	English	Journal paper	Review	This paper discussed the history and current status of PC in Turkey.	The first institution that provided long-standing PC was ‘Cancer Care Centres’ established by the Turkish Oncology Foundation in Yeşilköy İstanbul. These centres provided service between 1993 and 1997. However, services were not reimbursed within the social security system. In 2006. ’Hacettepe Hope House’ was founded to provide a minimal nursing and accommodation service to patients with cancer for a few weeks during their treatment and the following period. The first PC unit, and ‘Palliative Care Practice and Research Centre’ was established in Ege University. By January 2016, there were 148 palliatives care centres that provide services with a registered bed capacity of 1,672 in 29 Healthcare Regions.
Guzelant *et al* [92]	English	Journal paper	Tool validation	To evaluate the validity and reliability of the Turkish version of the European Organization for Research and Treatment of Cancer Quality of Life Core Questionnaire version 2.0 (EORTC QLQ-C30).	The EORTC QLQ-C30 is a valid and reliable instrument for Turkish lung cancer patients and can be used in clinical studies but needs supporting by the reference data on the Quality of Life of the Turkish population.
Eyigor [60]	English	Journal paper	Review	In this review effects of exercise in PC for cancer patients.	Fatigue is one of the most frequent symptoms in PC patients. And it is associated with anxiety–depression, pain, dyspnoea, insomnia, anorexia, nausea and drowsiness that reduces daily activities and affects Quality of Life (QoL) of patients. Physical therapy and rehabilitation practices can have a positive impact on symptoms, functional capacity and QoL.
Isikhan [282]	English	Journal paper	Review	In this review, coping strategies used by advanced cancer patients were described.	Cancer persons report using more avoidant coping strategies such as escape/avoidance denial or behavioural disengagement. The use of such strategies is associated with increased psychological distress and may be a risk factor for adverse responses to illness. PC staff who are may identify the need for support and assistance with coping strategies.
Yeşilbalkan *et al* [94]	English	Journal paper	Tool validation	To test the validity and reliability of the Edmonton Symptom Assessment Scale for Turkish cancer patients.	The Turkish version of the Edmonton Symptom Assessment Scale was determined to be a valid and reliable tool for use in Turkish individuals with cancer.
Yildirim [99]	English	Journal paper	Tool validation	To evaluate its reliability and validity of the Memorial Symptom Assessment Scale.	The Memorial Symptom Assessment Scale is a reliable and valid instrument for the use in the Turkish cancer patients for comprehensive symptom assessment in planning nursing care.
Sahin and Tan [103]	English	Journal paper	Cross-sectional	To determine whether Turkish patients with cancer and their caregivers differed in feelings of loneliness and depression.	This study enrolled 60 caregivers and 60 cancer patients admitted at the Oncology and Hematology Department of a Teaching Hospital. 70% of patients and 63% of caregivers reported a high level of loneliness. The average loneliness score for patients was more than caregivers’ scores. Serious depressive symptoms were experienced by 57% of patients and 71% of caregivers. Levels of perceived social support from family differed between patients and caregivers. Both groups showed a strong inverse relationship existed between depression, loneliness and perceived social support from family.
Akyar *et al* [84]	English	Journal paper	Qualitative/semi-structured interviews	Adaptation of the ENABLE (Educate, Nurture, Advise, Before Life Ends) evidence-based early PC model for Turkish family caregivers of cancer patients.	Semi-structured interviews were conducted with 25 primary family caregivers. The caregivers described the impact of cancer on their daily lives and responsibilities in the areas of physical, psychological, work, social and family life. Caregivers emphasised their needs for information about symptoms, physical care, cancer pathology and prognosis. Regarding the ENABLE model of early concurrent PC, participants wanted in-person training with educational material that should be simple and focused on disease information, psychological support, caring, nutrition and acquiring community services.
Kabalak *et al* [23]	English	Journal paper	Review	This article reports the structure and function of ‘Comprehensive Palliative Care Center’ in the State Hospital.	The multidisciplinary team is involved in patient management. There is room for training, physical activity and recreation activities. For spiritual care, preachers discuss the philosophy of Islam on life and death issues. The home care team visits discharged patients to record the vitals, wound care, nutrition and ventilation support. Due to the ambiguity of policies regarding PC, a ‘Long Term Intensive Care Units’ is also opened in the hospital.
Yildirim *et al* [70]	English	Journal paper	Cross-sectional	To examine information about the knowledge and attitudes of Turkish oncology nurses regarding cancer pain management.	Of the 68 oncology nurses, only 35.5% responded correctly regarding cancer pain management. The nurses’ pain management knowledge was positively correlated to the length of working experience in the oncology unit.
Ozveren and Kirca [254]	English	Journal paper	Cross-sectional	To determine the influence of PC training on the perception levels of nurses regarding spiritual care.	55.7% of the nursing students stated that they had never heard of spiritual care, and 81.4% had not obtained any information regarding spiritual care. The point average of pretest–posttest intervention using spirituality and spiritual care grading scale found that the perception of the student regarding spirituality and spiritual support was increased significantly after the training.
Okan *et al* [255]	English	Journal paper	Cross-sectional	To investigate whether a cultural mourning ritual, the ‘First Feast’, can be used by PC teams to ease the grief response of the deceased patient’s relatives.	The study included 427 relatives of PC patients in Tokat province. A total of 76.8% participants were from the Tokat and 77.8% performed the First Feast tradition. About 91% of the participants acknowledged that the tradition helped to ease the grief response of the relatives and might be a useful auxiliary method for PC teams to help grieving families.
Metin and Demirci [259]	English	Journal paper	Cross-sectional	This study aimed to investigate the effect of health literacy (HL) of the caregiver on the care results, including mortality, of 240 PC patients.	About 19.50% of the patient caregivers had adequate HL using the Health Literacy Survey – Europe Union. Caregivers had difficulty to access information of what to do in case of emergency. Prevalence of bedsore occurrence in patients whose caregiver has ‘inadequate’ HL is higher than patients whose caregiver has ‘adequate’ HL. Prevalence of ‘inadequate HL’ of the caregiver was higher among patient who died with-in 3 months after inclusion in the study.
Dagli *et al* [206]	English	Journal paper	Cost analysis	To evaluate the nosocomial infections and to understand factors affecting the cost of used antibiotics among 113 patients in the PC unit of the Hospital.	Nosocomial infections were observed among 74.3% of the cases, antibiotics were used in 92.0% of patients. The mean duration of antibiotic use was 23.13 ± 18.06 days, and the average antibiotic cost was 2,009.72 ± 2,153.37 TL. Length of stay, male sex, presence of decubitus ulcers, tracheostomy, enteral and parenteral nutrition significantly increased antibiotic cost. Antibiotic cost and mortality were found unrelated.
Bar-Sela *et al* [283]	English	Journal paper	Cross-sectional	To study the attitudes of oncology nurses and physicians toward spiritual care provision, in relation to Middle Eastern culture and Human Development Index.	773 responses from 14 countries were analysed (168 from Turkey). Over 63% of respondents positively viewed items such as spiritual history taking, referrals and encouraging patients in their spirituality. While significantly more, over 76%, did so among respondents from very high HDI countries. Only 42%–45% overall were positively inclined toward praying with patients. Overall respondents in lower HDI countries expressed significantly more positive views.
Bagcivan *et al* [112]	English	Journal paper	Tool validation	To determine the psychometric properties of the newly translated Functional Assessment of Chronic Illness Therapy Palliative Care (FACIT-Pal) scale for Turkish-speaking cancer patients-FACIT-Pal-TR.	The FACIT-Pal-TR demonstrates strong reliability and validity for evaluating PC specific QoL in Turkish cancer patients.
Saygili and Celik [207]	English	Journal paper	Cost analysis	To evaluate the cost‐effectiveness of three PC models	Three alternative PC models, namely; Comprehensive Palliative Care Center (CPCC), hospital inpatient services (HIS) and home healthcare (HHC) for cancer patients were analysed. From a societal perspective, PC services provided by the HIS model was found to be more cost‐effective than the CPCC model. From a patient perspective, HHC was found to be more cost‐effective compared to the other two models.
Yildiz *et al* [113]	English	Journal paper	Tool validation	To determine the validity and reliability of the Karnofsky Performance Scale (KPS) in cancer patients receiving PC.	KPS is a reliable scale for Turkish cancer patients in PC settings.
Akdeniz *et al* [75]	English	Journal paper	Interventional	Effect of web-based paediatric PC education on nursing students’ knowledge level and practices.	A statistically significant difference was observed between the pretest and posttest scores of the students given web‐based paediatric PC education (intervention group) and the control groups regarding the PC knowledge level and self‐reported PC practices.
Temelli and Cerit [253]	English	Journal paper	Qualitative/interviews	Interviews with PC nurses to identifying their perceptions about death and determining PC practices.	The palliative nurses had empathy when they met death in the unit where they worked. They perceive death as a natural and inevitable process and that as long as their working period increases, they become desensitised. They thought that death was a salvation for the patient. The nurses performed PC self-care needs, pain relief, prevention and management of pressure wounds.
Özdemir *et al* [258]	English	Journal paper	Cross-sectional	Psychosocial problems and spiritual coping styles of the 78 family caregivers of patients receiving PC.	The mean anxiety and depression scores using the Hospital Anxiety Depression Scale were 10.86 ± 4.30 and 9.38 ± 3.66, respectively. The mean positive and negative results of the Religious Coping Methods Scale were 25.31 ± 3.85 and 10.32 ± 3.38, respectively.
Kocatepe *et al* [114]	English	Journal paper	Tool validation	To examine the validity and reliability of the Turkish version of the Palliative Care Outcome Scale (POS).	The Turkish version of the POS a valid and reliable tool to be used for assessing the needs of PC patients. The Cronbach’s alpha reliability coefficients were determined as 0.64 for the patient questionnaire, 0.73 for the carer questionnaire and 0.68 for the staff questionnaire.
Tertemiz and Tuyluoglu [87]	English	Journal paper	Cross-sectional	To reveal the differences in the signs of burnout and stress between PC workers and those working in neurology and internal disease clinics.	Emotional burnout and desensitisation scores were found to be elevated, and personal success scores were low in both groups. The Beck Anxiety Inventory revealed moderate anxiety in both groups, while cognitive-sensorial, physiological and pain complaints, as well as signs of stress, were more pronounced among workers in internal disease and neurology clinics.
Clark *et al* [35]	English	Journal paper	Survey	The objective of this study was to describe current levels of global PC development and report on changes since 2006.	Based on the survey indicators the status of PC in Turkey was as follows: 2006; Category 2: Capacity-building PC activity 2011; Category 3b: Generalised PC provision 2017; Category 3a: Isolated PC provision
Arias-Casais *et al* [136]	English	Journal paper	Survey	To conduct the first regional assessment of paediatric PC development and Provision.	Number of providing Paediatric Palliative Care service PPC identified in Turkey in 2019 were as follows: Hospices services = 01 Home-care services = 00 Hospital services = 04
Yalcin *et al* [284]	English	Journal paper	Qualitative/expert panel	Statement by a panel of medical oncologists to provide guidance addressing nutritional aspects of cancer care.	Participating experts agreed on the use of liquid and high energy-dense oral nutritional supplements to enable better patient compliance, improved lean body mass, functional status and quality of life, as well as better tolerance to antineoplastic treatment.
Irmak *et al* [90]	English	Journal paper	Cross-sectional	Frequency of complementary and alternative medicine (CAM) therapies among cancer patients and to evaluate their quality of life.	Among 211 patients, 46.4% were CAM users. The most commonly used CAM therapy was herbal products. The rate of CAM use was higher among the patients with a low education level. No statistically significant difference was found between the quality-of-life scores of the CAM user and non-user patients.
Ozdemir *et al* [139]	English	Journal paper	Retrospective /cross-sectional	The role and contribution of hospital-based home health services regarding the respiratory illness.	The MoH data concerning HHS for respiratory diseases between 2011 and 2017 showed that the number of house visits concerning pulmonary disorders (COPD, lung cancer) increased nearly ten times, but hospitalisation due to respiratory diseases decreased (13.5% in 2011 to 12.9% in 2017).
Sarıcam *et al* [86]	English	Journal paper	Retrospective /Cross-sectional	Prevalence of neurological symptoms in cancer patients followed up in a PC centre.	The most common neurological symptoms were pain, insomnia, delirium, seizures, depression and paresis (27.1%, 17.2%, 15.1%, 13.5%, 11.5%), respectively. The pain was most commonly observed in genitourinary and gynaecologic cancers (72.4%) and gastrointestinal cancers (69.3%). Paresis and seizures were significantly higher in patients with head and neck cancers.
Baykara *et al* [220]	English	Journal paper	Cross-sectional	The opinions and attitudes of intensive care unit (ICU) physicians regarding End of Life (EoL) decisions, for both their patients and themselves.	Religious beliefs had no effect on the physicians’ acceptance of do-not-resuscitate (DNR)/do-not-intubate (DNI) orders for terminally ill patients. The medical experience and proportion of terminally ill patients in the ICU appears to affect physicians’ attitudes to EOL decisions. The younger physicians (30–39 years) were more likely to prefer the ‘only DNR’ option compared with physicians aged 40–49 years for themselves.
Centeno *et al* [133]	English	Journal paper	Mixed method; literature review, qualitative and quantitative survey	Facts and indicators on PC development in 52 countries of the WHO European region.	Provision of specific PC services were 0.3/million population. The development of paediatric PC specific resources was at level 1 (unspecific resources). The provision of specialist PC beds was 241 and there was no PC physicians working full time.
Guclu *et al* [221]	English	Journal paper	Cross-sectional	Symptoms of depression and anxiety of caregivers of patients hospitalised in the extensive PC centre.	The risk of depression was identified as 43% according to the Beck anxiety scale (BAS), and as 91% according to the hospital anxiety and depression scale (HADS). The anxiety rate was 66% according to the BAS. Among these, 36% demonstrated mild anxiety, 30% demonstrated moderate anxiety and 32% demonstrated severe anxiety. According to HADS, the risk for anxiety was 72%.
Turkoglu and Kilic [238]	English	Journal paper	Cross-sectional	The effects of caring burden of family caregivers of cancer patients on their quality of life in the East of Turkey.	The mean score of the burden of caregivers was 36.6 ± 11.2 and their mean score of Caregiver Quality of Life Index-Cancer was 81.4 ± 17.3. There was a negative relationship between caring burdens and the quality of life (*p* < 0.001). Descriptive characteristics, caring-related properties and caring burden variables were all significant predictors of the quality of life. The caregivers should take support by training about providing care.
Vardar Yağlı *et al* [62]	English	Journal paper	Interventional	To compare the effects of aerobic exercise training and yoga on the functional capacity, peripheral muscle strength, quality of life and fatigue in breast cancer survivors.	There were statistically significant increases in peripheral muscle strength, the 6-Minute Walk Test distance and the perception of QOL in both groups. Additionally, the group with aerobic exercise and yoga showed marked improvement compared with the aerobic exercise group in fatigue perception.
Gültekin *et al* [27]	Turkish	Journal paper	Review	This review article explores the status of PC in the year 2010 in Turkey.	There were nine PC services across the country and seven were located in university hospitals. There were 72 pain centres of these, 35 were in located governmental hospitals and 31 were in university hospitals while 6 were in the private sector. Approximately 40% of pain centres were located in Ankara and Istanbul. It was found that morphine consumption rates per capita were relatively lower and the availability of different types of opioid analgesics was limited.
Saygili and Celik *et al* [212]	Turkish	Journal paper	Case–control	To evaluate the effect of PC on the symptom level assessment and satisfaction of patients diagnosed with cancer.	The PC provided to 60 cancer patients at the PC centre was less effective in reducing symptom levels (fatigue, nausea and dyspnoea) compared with the 59 cancer patients who received general care services at a public hospital. However, the mean satisfaction of the patients who received services at the PCC was significantly higher.
Ozcelik *et al* [191]	Turkish	Journal paper	Review	This review discussed case management-based multidiscıplinary care protocol in the PC of cancer patients.	Care coordination and case management are essential of a multidisciplinary team approach to provide effective and quality care, to patients and families. A lot of care guidelines and protocols are developed by a team of experts to contribute to the PC activities and could be used.
Şenel *et al* [123]	Turkish	Journal paper	Cross-sectional	The objective of the study was to describe patterns of opioid use in the PC clinic of a training hospital.	Out of 418 cancer patients, 65% were prescribed strong opioids, 26% of patients were with weak opioids. The daily oral morphine equivalent dose per patient was 172 ± 58 mg. The indications for opioid use were pain (61%), dyspnoea (19%) and both dyspnoea and pain (20%).
Tekin [285]	Turkish	Journal paper	Case report	This is a case report of an old lady who had given institutional care at the terminal period of her lung cancer.	In this report, the interdisciplinary geriatric teamwork including the role of the family physician was evaluated.
Uysal *et al* [117]	Turkish	Journal paper	Interventional	To evaluate the effect of PC on patient symptoms by recording changes during the first week after hospital admission.	Among 108 patients admitted to the PC unit, 50% rated pain intensity at the level of 5 upon admission, and in follow-up, only 6.5% of patients had pain intensity at 5 on the 7th day of admission. The mean values of the symptoms of pain, insomnia, loss of appetite and status of well-being decreased significantly on the third day of admission compared to the time of admission to the hospital. However, no significant difference was found among the symptoms of nausea, anxiety, shortness of breath and constipation after 7 days of admission.
Yildirim [286]	Turkish	Journal paper	Review	To provide information about complementary therapies used by PC cancer patients.	Complementary medicine has become an important aspect of palliative cancer care. Acupuncture, aromatherapy, homeopathy, hypnotherapy, massage, reflexology, relaxation techniques and spiritual healing are frequently used forms of treatment.
Çavdar [287]	Turkish	Journal paper	Review	This article focuses on the physiological, psychological, spiritual and sexual needs of patients in the terminal period.	The needs and rights of dying patients and their family members we discussed. The supporting approaches by healthcare staff might reduce the burden to caregivers and improve quality of life in the last hours.
Kabalak [288]	Turkish	Journal paper	Review	This paper status of PC in Turkey in the year 2014.	Need for renewing existing PC programmes within the framework of the country’s current demographic, economic, socio-cultural profile. The system should be renewed according to its belief structure. A multidisciplinary and multifaceted approach is needed for the improvements.
Ay and Gençtürk [69]	Turkish	Journal paper	Qualitative/focus group discussion	Midwifery student’s opinions related to death, terminal period and palliative care.	Midwifery students feel helplessness and inadequacy in training to face terminal phase emotional support to the dying patients and their caregivers. Adding PC education to the midwifery curriculum is necessary to improve the quality of care at the end of life.
Demir [289]	Turkish	Journal paper	Review	This article was aimed to draw attention to ethical dilemmas that arise with a PC need.	The paucity of trained and experienced PC healthcare professionals in Turkey. PC ethical issues are necessary for the training of healthcare providers.
Isikhan [290]	Turkish	Journal paper	Review	In this review, the choices of the place of death of cancer patients and the factors that affect these choices are discussed.	For cancer patients and their caregivers in reaching the period of dying in peace and dignity, the choice of place of death is important in developing the quality of life during dying. Hospice and home care are needed to developed and PC team members and caregivers are needed to be trained.
Zengin and Büyükbayram [235]	Turkish	Journal paper	Cross-sectional	Satisfaction levels of the cancer patients in the PC unit in terms of nursing care.	The satisfaction of the patients in terms of nursing was found to be above average. It can be suggested to evaluate the patient satisfaction in with nursing care and take steps to increase satisfaction.
Saruç [63]	Turkish	Journal paper	Review	Role of social workers in PC.	In this study, the roles of the social worker in PC and the professional standards established by the National Association of Social Workers for social workers in palliative and life care were discussed.
Koç *et al* [239]	Turkish	Journal paper	Cross-sectional	Factors effecting the caregiving load of caregivers for patients with cancer.	71.2% of patients with breast cancer have fatigue and 48.2% have nausea. The care taking load scale of care takers was average 55.6 ± 13.0, 48.8% of them have medium care load and 38.8% of them have heavy care load.
Kurt *et al* [242]	Turkish	Journal paper	Cross-sectional	Quality of life of caregivers of patients with cancer and the affecting factors.	Most (70.9%) of the caregivers were female, and 55.5% expressed that there was no one else for caring the patient, 92.8% of the caregivers were family member or relative of the patient. The mean Caregiver Quality of Life Index-Cancer total score of the caregivers was 72.16 ± 14.07. The quality of life of the caregivers who had no another caregiver, no social insurance and poor income status was found to be statistically lower (*p* < 0.05).
Akyar *et al* [243]	Turkish	Journal paper	Cross-sectional	Outcomes and changes in their life due to caregiving for elderly patients with cancer.	Caregivers reported negative impact in ‘physical health, coping strategies, relationship with friends, energy level, emotional wellness, time for social activities and physical functioning’ and positive impact in ‘relationship with the patient’.
Bİlgin *et al* [182]	Turkish	Journal paper	Cross-sectional	Levels of care dependence and the factors affecting care dependence of inpatients in nephrology and oncology clinics.	It was found that 60.5% of the oncology patients were care-dependent. Education level (*p* = 0.006), economic status (*p* < 0.001), cane use (*p* < 0.001), hearing problem (*p* < 0.001), speech problem (*p* < 0.001), chewing problem (*p* < 0.001) and walking problem (*p* < 0.001) affected care dependency significantly.
Avcı [222]	Turkish	Journal paper	Cross-sectional	Relationship between the Nutrition Risk Screening (NRS-2002 scores) and haematologic inflammation markers.	The neutrophil/lymphocyte ratio (NLR), platelet/lymphocyte ratio (PLR) are in correlation with NRS-2002 scores. These comparisons were made for the first time in the area of PC centres.
Arslan *et al* [170]	Turkish	Journal paper	Cross-sectional retrospective file search descriptive	Effectiveness of palliative pain management in cancer patients.	A significant reduction in pain scores with effective analgesic treatment was observed. The mean VAS pain scores of the patients recorded during hospitalisation was 5.2 ± 2.6 and the mean of these values at discharge was 2.2 ± 1.8. Obstacles to the treatment of pain; the doctors who are lack of information about pain treatment, fears about drug addiction, the patients who hide their symptoms and ignoring to symptom control as can be listed.
Elçigil [55]	Turkish	Journal paper	Review	Nursing care for PC.	An effective PC focuses on the patient and the family and requires a multidisciplinary approach. Doctors, nurses, social worker and religious functionaries play a role in PC. A nurse involved in a PC team should be able to make a comprehensive evaluation of patients and their families’ needs. She should have effective communication skills, listen to them carefully, respect patients and their families’ knowledge and decisions. The nurse provides support and care for all family members of a patient. The nurse in a special PC team should be aware of their abilities and limitations. She should always update their knowledge and skills.
Keser *et al* [61]	Turkish	Journal paper	Review	Physiotherapy and rehabilitation for patients with cancer.	277 (54.5%) patients received in-patient oncological physiotherapy service while 231 (45.5%) received out-patient oncological physiotherapy service. Five training programmes were organised for the patients and their relatives.
Gelin and Ulus [172]	Turkish	Journal paper	Cross-sectional	Quality of life of the patients receiving chemotherapy and the influencing factors.	It has been observed that there is no difference between the men and women in terms of their health condition, social life and personal status. The scores of functional state of health of men are higher than women while subscale scores of emotional conditions of women are higher than men. There is a positive correlation between the income and social life of patients and their functional state of health. The functional state of the health of the patients living with their family and having no financial problems was higher compared to those having financial problems.
Tunçel *et al* [240]	Turkish	Journal paper	Cross-sectional	Nurses’ burnout in oncology hospital care unit.	High levels of emotional exhaustion in 82% and depersonalisation in 51.4% of nurses was determined. Personal accomplishment was higher at 80%. Mild to moderate emotional state and mild anxiety was revealed. Years in profession, insufficient salary, finding the profession suitable for herself, choosing the PC unit willingly, satisfaction of work environment and social activity were associated with burnout (*p* ≤ 0.05).
Karabulutlu *et al* [245]	Turkish	Journal paper	Cross-sectional	Sleep quality, anxiety and depression levels and affecting factors in cancer caregivers.	A statistically significant positive correlation was found between sleep quality score and anxiety and depression levels. 88.7% of caregivers had poor sleep quality and the average total sleep quality was 9.87 ± 3.95. The rate of anxiety was 46% and risk of depression was 72% among caregivers.
Bağtatlı and Eşer [78]	Turkish	Journal paper	Cross-sectional	Pain assessments of the nurses and factors affecting their pain assessments.	The nurses working in oncology clinics mostly evaluate verbal expressions of patients for assessing pain and none of them used pain scale, the level of assessed pain was lower than the self-report of cancer patients. Nurses’ education level, training about pain and working duration in oncology clinic affected their pain assessment.
Yıldırım and Gürkan [189]	Turkish	Journal paper	Cross-sectional	The influence of music on anxiety level of patients and chemotherapy side effects.	The findings indicated that, music has a meaningful relation with respect to anxiety states of the sample group (*p* > 0.05), and had no such a relation for the side effects of chemotherapy (*p* < 0.001).
Kart *et al* [199]	Turkish	Journal paper	Cross-sectional	Medical cost of the terminally ill cancer patients hospitalised in the medical intensive care unit (ICU) of university hospital.	Medical cost of the 15 patients died in the intensive care unit and two patients discharged with home mechanical ventilator support was 208.200,640/TL. They were hospitalised for 233 bed/days. The number of beds occupied by these terminal phase patients in this period was considered to be high. Foundation of PC units or hospice service with necessary law evaluations is very useful and important not only for optimal use of limited financial sources but also for comfort of patient and relatives.
Ateşçi *et al* [224]	Turkish	Journal paper	Cross-sectional	The prevalence of psychiatric morbidity among cancer patients and the factors predicting psychiatric morbidity.	28.7% of cancer patients were found to have the diagnostic and statistical manual (DSM-IV) Axis I diagnosis. The most common diagnoses were adjustment disorder with depressed mood (14%) and major depressive disorder (11.3%). Female gender, awareness of the diagnosis of cancer, history of previous premorbid psychiatric disorders and stress factors were correlated with psychiatric morbidity
Usta Yeşilbalkan *et al* [173]	Turkish	Journal paper	Cross-sectional	She symptoms due to chemotherapy and their effects on the quality of life.	During course of the treatment the patients feel ‘a little’ anorexia (39.8 %) and ‘a little’ anger (45.6%). A weak and negative relationship was found between patients’ overall quality of life and physical (*r* = −0.2292) and psychological (*r* = −0.2379) symptoms that they encountered throughout the course of treatment.
Üzelli Yılmaz *et al* [241]	Turkish	Journal paper	Cross-sectional	Quality of nursing care in a PC clinic.	The average score of Care Behaviors-24 Scale (BDI-24) was found as 5.59 ± 0.15 for nurses and 5.10 ± 0.15 for patients. There was no statistically significant difference between the average of BDI-24 total points and subscale scores of nurses according to their gender, education status, working duration in PC clinic and weekly.
Karabuğa Yakar and Pinar [216]	Turkish	Journal paper	Cross-sectional	Quality of life and effecting factors of 120 caregivers of patients with cancer.	The quality of life of the caregivers assessed by Caregiver Quality of Life Index-Cancer was quite low (80.6 ± 19.3). They could not keep-up with daily life responsibilities (53.3%), and they had problems with their working life (30%), relationship within the family (15%) and spouses (45%).
Aydoğan and Uygun [291]	Turkish	Journal paper	Review	Palliative treatments in cancer patients.	PC should be part of comprehensive cancer care, rather than a separate speciality.
Güngör Tavşanlı *et al* [65]	Turkish	Journal paper	Cross-sectional	The feelings and attitudes of nurses and nursing students caring for patients with cancer.	Study group consisted students (77.7%) whom 75% were college freshman and 52.9% were graduates of non-vocational high school, and nurses. The item ‘I think it is necessary for cancer patients to know about their illness’ was answered positively by 91.1% of fourth-year students. The item ‘I think it is necessary to tell patients the whole truth concerning their treatment’ was answered positively by 67.9% of fourth-year students
Sarıhan *et al* [292]	Turkish	Journal paper	Review	The importance of cancer pain as a social problem and pain management.	Appropriate pain management with World Health Organization analgesic ladder can improve patients’ quality of life and pain relief can be achieved in about 80% of patients.
İnci and Öz [293]	Turkish	Journal paper	Review	PC and death anxiety.	In terms of the end of life, it is expected that the nurse stands by patient’s family to help them in sustaining their psychosocial wellness. In order to meet this expectation, nurses should get a qualitative training for end of life care along with good communication skills and coping strategies.
Uslu Sahan and Terzioğlu [294]	Turkish	Journal paper	Review	PC education and organisation in the World and Turkey.	In Turkey, the PC activities are supported with a number of efforts for its integration with the national health system. To overcome the problems experienced in the PC services, the Cancer Control Department under the Ministry of Health has designed a nurse based and community focused project called ‘PALLIA-TURK’. A considerable improvement has been scored in the PC training and organisation within the framework of this project, and such improvements are expected to increase rapidly and steadily.
Avcı and Avcı [171]	Turkish	Journal paper	Review	The effects of nutritional status of the cancer patients in the PC unit (PCU) on mortality and duration of hospitalisation.	There was a statistically significant difference between the median hospitalisation days of patients admitted from home and from hospital (11 days versus 22 days) (*p* =0.001) in PCU. Their median survival time for both groups were 87.5 days and 9 days, respectively (*p* = 0.017). The death rates were 29.5% for patients admitted from home and 70.5% for patients admitted from other units of hospital (*p* = 0.002). The NRS-2002 scores of the cancer patients who were followed up at the palliative unit were correlated with the age of the patients (*r* = 0.365, *p* = 0.003)
Can *et al* [124]	Turkish	Journal paper	Cross-sectional	Pain palliation for lung cancer patients.	Of 72 lung cancer patients 27.8% had admitted with the only complaint of pain. The median duration of hospital stay was 7 (2–45) days. Among all the 18% of patients were not given any analgesics; 49% had second line drug added and 50% had received third line treatment. Hospitalisation indication and discharge status of the patients were statistically significant associated with analgesic use.
Benli and Sunay [141]	Turkish	Journal paper	Cross-sectional	Coordination of a PC unit (PCU) and home health care (HHC) in a university hospital and services of PCU.	During 2016, 149 patients were hospitalised in PCU. Average hospitalisation duration was 14 ± 12 (1–79) days. Neurological diseases and malignancies were the most underlying diseases and nutritional support and decubitus ulcers were the frequent etiologies for hospitalisation. Most (78%) of the patients were bedridden. Mortality rate was 12% for 1 year. Performance scores of the patients at discharge time were higher than scores at hospitalisation (*p* < 0.001).
Babaoğlu and Öz [223]	Turkish	Journal paper	Cross-sectional	Psychological and social problems of the spouses of terminal cancer patients.	The most frequent psychological problem was depressive affect and the most frequent social problem was impaired social interactions. Physiological and social problems are related with each other. Spiritual distress and hopelessness were related with ineffective coping and ineffective role performance, and ineffective coping was related with impaired home maintenance and caregiver role strain. The following significant relations are found for the social problems. These relations are between; impaired home maintenance, social interaction and role performance, deficient diversional activity, social isolation. At the same time it have been observed a relationship between ineffective role performance and social isolation, too.
Gemalmaz and Avşar [257]	Turkish	Journal paper	Qualitative	Feelings of patients after the news of cancer and changes of life.	Patients easily shared their diagnosis of cancer with their friends, but it was hard with family. They were overwhelmed by intense concern of the family. The support of the family in accepting the cancer after diagnosis was positive and they also indicated they tend to feel spirituality more intensely.
Akgün and Akan [54]	Turkish	Journal paper	Review	Paediatric PC.	PC in children contains the assessment and handling of the childrens’ and family’s physical, psychosocial and spiritual needs and also symptom control and support to family during the illness and mourning process.
Okçin [56]	Turkish	Journal paper	Qualitative	Professional life experiences of nurses in PC and oncology clinics.	After the descriptive and interpretative analysis of the data, three main themes and seven sub-themes were created. Themes of challenges were fatigue, difficulty in coping/burnout and communication problems. Themes of gains were professionalisation/satisfaction, meaning of life and death and team harmony. Coping appeared as a sub-theme of field-specific experience.
Kurşun *et al* [125]	Turkish	Journal paper	Cross-sectional	Characteristics of 1,736 cancer patients and pain management.	The adjuvant pain medications were antidepressants, corticosteroids, anticonvulsants, neuroleptics, benzodiazepines, local anesthetics, bisphosphonates and calcitonin and their rate of usage were 61.0%, 7.1%, 4.0%, 4.0%, 1.6%,12.3%,1.2% and 2.8%, respectively.
Orhan *et al* [126]	Turkish	Journal paper	Cross- sectional	Pain management according to the World Health Organization analgesic ladder treatment and other treatment modalities in cancer patients.	WHO analgesic ladder was used for treatment algorithm in 87.5% of patients and invasive techniques were needed in 12 % of patients. The number of successfully treated patients in step I, II and III were 11%, 73% and 14%, respectively. Also, 43% of patients used anticonvulsants or neuroleptics and 81% of patients used antidepressants. Non-invasive or invasive treatment modalities had to be added in 7% of patients to augment the WHO analgesic ladder treatment.
Aygencel and Türkoğlu [208]	Turkish	Journal paper	Retrospective	General characteristics and costs of terminal-stage patients in a medical intensive care unit (ICU).	Eight percent of patients admitted to the ICU during the study period were terminal-stage patients. The median age was 63 years, 52% of patients were male and 77% of patients were terminal-stage cancer patients. Despite full support therapy, only 4.8% of the patients were discharged. The median cost was 2841 TL, and the total cost was 581,353.2 TL.
Genç *et al* [169]	Turkish	Journal paper	Cross-sectional	Nonpharmacological methods for the pain management of cancer patients.	The average age of the patients participating in the study was 58. Of the patients, 53.7% were female, 53.7% were literate-primary school graduates and 29.3% had breast cancer. Of the patients, 54.4% said they had severe pain, and 82.9% said they used painkillers. It was found that 87% of the patients prayed for the pain relief, 63.4% massage the pain area, 59.3% read the Qur’an and 58.5% used breathing exercises.
Bal Yılmaz *et al* [146]	Turkish	Journal paper	Cross-sectional	Parental knowledge of cancer-related symptoms and their attitude regarding fatigue in children treated for cancer.	Parents observed severe alopecia in 48.3%, fatigue in 49.4%, nausea-vomiting in 34.8%, anxiety in 24.7%, moderate taste change in 23.6%; constipation/diarrhoea in 28.1%, pain in 7%, sleeping difficulty in 12.4% and rare respiratory distress in 7.9%. Parents reported cancer-related fatigue severely affecting their children’s school activity in 43.8%, friendships in 39.3%, psychology in 37.1%, play activity in 36%, energy in 29.2% and family relationships in 28.1%.
Peker *et al* [77]	Turkish	Journal paper	Cross- sectional	Opinions, knowledge and attitudes of doctors on cancer pain management in a university hospital.	Two thirds of the doctors feel themselves ‘insufficient’ in cancer pain management. Insufficiency feeling was more prominent in tasks requiring knowledge, skill, education and experience about opioid use. Most of the doctors believe that barriers originating from health professionals and systems are more important than the ones resulting from patients and it was necessary to give high priority to pain management than treatment of cancer; but still half of them report that legal regulations have some influence on opioid prescription; and almost three quarters of them believe that opioid use may cause high rates of psychological addiction or abuse.
Bilen *et al* [127]	Turkish	Journal paper	Interventional	The frequency of break-through pain (BP) and the efficiency of oral transmucosal fentanyl citrate (OTFC) for the treatment.	The frequency of BP was found as 63.7% in our study. The appropriate OTFC dose in the titration phase was 200 μg for 10 patients, 400 μg for 21 patients and 800 μg for 17 patients. In four patients OTFC failed to control their BP attacks. An appropriate dose of OTFC was found effective in 81.1% of BP attacks. A significant difference was determined in VAS scores before and after OTFC use (*p* < 0.001). The average duration effect of OTFC was determined as 17.7 ± 8.28 minutes. No serious side effect was reported in any patient.
Yılmaz and Atay [66]	Turkish	Journal paper	Cross-sectional	Pain management knowledge of nursing students.	Nursing students took notice of pain complaint of their patients (86) and they observed pain in the patients in postoperative period (51%). Half of the students (48%) defined pain as a discomforting condition. In the sample case given, 63.5% of the students defined the nursing interventions in order of assessing the pain with scale, using non-pharmacological methods, and giving analgesic drugs. And 36% of the students stated giving analgesics according to pain scale was routine nursing interventions.
Işık *et al* [138]	Turkish	Journal paper	Cross-sectional	Profile of patients using home care health services and evaluation of the provided service.	‘Home Health Care Services’ was first introduced in as a part of Health Transformation Programme brought health care to home. It was aimed to be provided regular and quality health care to patients such as elderly, bedridden, disabled or cancer patients, people who have chronic diseases like joint-muscle diseases and need care post-operative by professional medical team in their own home environment with this programme. In this study, the aim was to evaluate if the system met the needs of patients and search the quality of home health care service in Kırıkkale. Patients stated they were satisfied from behaviour of personnel like their kindness and smiling faces (%4,0) and their attitudes when dealing with problems (3.97%) and competency (3.87%). But explanations of the personnel were inadequate (21.3%). Overall, home health care services provided in Kırıkkale meet the needs of patients.
Çivi *et al* [174]	Turkish	Journal paper	Cross-sectional	Depression in caregivers of the patients with cancer and factors affecting their quality of life.	According to the values of the inventory, 65.5% were normal, 24.5% mildly, 7.3% moderately and 2.7% severely depressed. The gender, occupation, education and marital status of the caregivers of the cancer patients did not affect the depression status (*p* > 0.05). When we compared the quality of life scores and depression status, there were significant differences in psychological health (*p* = 0.000), perception of overall health and the satisfaction from life (*p* = 0.002), general health and the quality of life (*p* = 0.008), physical health (*p* = 0.001) and environmental area (*p* = 0.025) while there was no statistically significant difference in social relationships (*p* = 0.089) between the cases with and without depression.
Kardaş Özdemir *et al* [244]	Turkish	Journal paper	Cross-sectional	Burden care of mothers of children with malignancy.	Average Zarit Burden Care of Scale scores of mothers were 21.29 ± 12.00. Care burden was related with income (*p* < 0.05). Health perceptions were statistically different before cancer and during caregiving period (*p* < 0.001). But, Burden Care of Scale scores of mothers was not very high.
Düzgün *et al* [225]	Turkish	Journal paper	Qualitative	Bereavement in caregivers of patients died in PC unit (PCU) and internal medicine intensive care unit (ICU).	As the patients’ quality of life increases with the service provided at the PC unit, the hope of recovery increases in caregivers. Most caregivers expect the death is more likely in ICU, that’s why we think hope level was higher in PCU.
Madenoğlu [295]	Turkish	Journal paper	Review	To review the health care on PC in Turkey.	It is important to prevent symptoms in PC. Both pharmacologic and nonpharmacologic methods should be used to provide a comfortable life for all patients.
Gültaş and Yılmaz [119]	English	Journal Paper	Cross-sectional	To determine challenges experienced by and quality of life of relatives of cancer patients requiring PC at home.	About 50% of care givers were women, 75% had difficulty fulfilling their responsibilities, 53.3% experienced problems in maintaining family relationships, 96.7% did not utilise home care services and 43% did not receive information about home care. The caregivers also had trouble managing pain, nausea/vomiting, defaecation and mobility (35%). Respondents’ quality of life was generally low.
Yildiz *et al* [271]	English	Letter to the editor	Qualitative/expert panel	Choosing Wisely® health initiative.	Choosing Wisely® health initiative was established by the American Board of Internal Medicine Foundation to advance a national dialogue on avoiding unnecessary medical tests, treatments and procedures in 2012. The Turkish Society of Internal Medicine (TSIM) has been working with the European Federation of Internal Medicine within the frame of the Choosing Wisely Project since January 2017. ‘Don’t delay palliative care” is one of the recommendations that scored the highest points among the members of TSIM.
Benli and Erbesler [49]	Turkish	Letter to editor	Review	Differences on comprehension and practice in PC in Turkey.	Working with PC services together with home care services will increase the efficiency of the service. When it is needed for physician consult for a patient in home care service, referring the patient to the PC service might improve care. Since family physicians have more comprehensive approach to their patients’ home care systems would benefit their contribution.
Guven *et al* [296]	English	Conference abstract	Cross-sectional	First 5 month experience of outpatient PC clinic at University hospital.	A total of 174 patients were seen. The chief complaints were pain (26%), nausea and vomiting (13%) and decreased feeding (9%). The most frequent interventions were intravenous hydration, analgesic and/or antiemetic administration and prescription. Thirty-three patients were referred for immediate hospitalisation. The overall mortality within the first 30 days after the first OPC visit was 17%.
Alkan *et al* [297]	English	Conference abstract	Cross-sectional	To evaluate the impact of the patient–physician relationship (PPR) on Fear of cancer recurrence (FCR).	There was a high level of FCR scores in 51% of 1,580 cancer survivors who were under remission. There was a negative correlation between PPR and FCR scores. In multivariate analysis; young age, being female, history of non-routine imaging and worse were associated with high levels of FCR.
Topkaya [97]	English	Conference abstract	Cross-sectional	To determine the PC needs of cancer patients and to examine the knowledge and expectations of the patients and their families about PC.	Among 110 patients and 110 patient’s families, 76.4% of the patients and 60% of their families were not aware of palliative/supportive care. Expectations of 99.1% patients and 97.3% of their families were to learn the treatment and care plan; 97.3% of the patients and 80% of the families wanted to be included and supported in the decisions making. 77.3% of the patients/85.5% of the families wants to be supported psychologically, whereas 77.3% of the patients/50% of the families needed religious support.
Eskigulek and Kav [118]	English	Conference abstract	Tool validation	To evaluate Turkish validity and reliability of The Patient Dignity Inventory (PDI) among PC patients.	Turkish version of the PDI is a valid and reliable instrument among PC patients Cronbach’s coefficient alpha for the PDI was 0.94 and test–retest reliability was *R* = 0.75.
Bagcivan *et al* [153]	English	Conference abstract	Cross-sectional	To examine temporal trends in the symptom experience of cancer patients presenting to an outpatient PC clinic.	Two hundred and thirty-eight cancer patients presenting to a PC outpatient. Patients’ most common symptoms were pain, fatigue, disturbed sleep and mild to severe depression.
Kütük *et al* [198]	Turkish	Conference abstract	Retrospective	Demographic and clinical data of cancer patients who received palliative chemotherapy in the last 6 months of their life.	About 28% of 262 patients had colorectal cancer, 10% breast cancer, 33.5% of them were bronchial cancer and 28.5% of them were other solid cancers. The mean age at death was 47, the male/female ratio was 145/117. Among all 42% of the patients had received palliative chemotherapy in the last month of life.
Akyar [266]	English	Oral presentation	Review	Palliative and supportive care in Turkey: literature review and the current status of research.	Bibliographic reviews of published PC literature in Turkey were presented.
Taçyıldız *et al* [128]	Turkish	Oral presentation	Cross-sectional	PC support and pain management in terminally ill patients.	Pain is an important problem that should not be ignored. Other problems we encounter in end-stage cancer patients are nausea-vomiting, nutritional problems, respiratory distress, depression, and chemotherapy side effects.
Palalı *et al* [175]	Turkish	Oral presentation	Cross-sectional	The frequency of pain in children: the effect of pain management and quality of life.	Pain management requires a multidisciplinary team approach. Oncology nurses should determine the need for pharmacological or non-pharmacological interventions after the patient is admitted. Pain assessment is required at least every 8 hours and more frequently after the painful interventions or treatment.
Savran *et al* [176]	Turkish	Oral presentation	Cross-sectional	The relationship between the quality of life of children with cancer and their parents’ health care satisfaction.	It was found that increasing the quality of life of the child increased the health care satisfaction of mothers. To increase the quality of life of the child, it is necessary to eliminate the symptoms experienced and plan nursing interventions for these symptoms control.
Çırpan Kantarcıoğlu *et al* [226]	Turkish	Oral presentation	Cross-sectional	Disease perception among adolescents diagnosed with cancer.	Adolescents’ negative beliefs should be determined and replaced with more positive cognitive content. It was reported that distressing symptoms such as pain required to be reduced as much as possible. Besides these, emotional states such as depression and anxiety should be treated.
Özdemir and Taşçı [185]	Turkish	Oral presentation	Interventional	Acupressure in fatigue of elderly people with cancer.	Acupressure can be recommended to elderly people with cancer as an easily applicable and tolerable method without serious side effects in reducing cancer-related fatigue.
Şen *et al* [186]	Turkish	Oral presentation	Interventional	Reflexology for pain, anxiety and Nausea.	A positive effect on pain, nausea and anxiety score after the application of reflexology was noted in approximately 100% of patients.
Gültaş and Yılmaz [298]	Turkish	Oral Presentation	Cross-sectional	Difficulties and quality of life experienced by caregivers of patients with cancer needing PC at home.	Full dependency rate was 26.7% in taking a bath and 23.3% in dressing and toilet needs. Most of the patients (61.7%) can take a bath, 63% can dress, 65% can meet their toilet needs, 71.7% can walk and 65% need help for meals. The total quality of life scale score was 49.7 ± 12.7 (min-max: 34–94) and accordingly, their quality of life was low.
Uğur *et al* [57]	Turkish	Oral presentation	Cross-sectional	The perceptions of nurses working in a university hospital towards PC and barriers of application.	Not realising the patient’s need for PC, lack of knowledge, lack of communication with families and patients, inadequacy of number of health professionals as well as PCUs and barriers in health policies are among the important deficiencies.
Kuşçu *et al* [156]	Turkish	Oral presentation	Cross-sectional	Hospitalisation indications and nursing care need of patients diagnosed with malignancy.	It has been observed that PC is very important in relieving the physical and psycho-social symptoms of patients with a curative treatment plan and nursing care need.
Koku F [248]	Turkish	Oral presentation	Cross-sectional	Nursing attitudes towards patient care in the terminal period.	In-service training should be given frequently for nurses to have knowledge and skills about the death symptoms and the care of the dying patients. Communication skills, knowledge of cultural differences and pain management are important for those working at end-of-life care units.
Özçelik *et al* [157]	Turkish	Oral presentation	Cross-sectional	Symptom distribution of advanced stage cancer patients and factors affecting them.	The total symptom levels of the patients were high, they experienced of pain and fatigue more intensely. Their appetite and well-being levels were low. It was determined that patients with low performance level experienced more intense pain and lethargy symptoms.
Kutlutürkan *et al* [177]	Turkish	Oral presentation	Cross-sectional	The practices of cancer patients regarding the problems that develop due to chemotherapy and its effect on the quality of life.	RAND 36-Item Short Form Health Survey -32 life scale scores of cancer patients were below average. The patients got the highest score from the physical function sub-scale and the lowest score from the social function sub-scale.
Yavuzşen *et al* [148]	Turkish	Oral presentation	Cross-sectional	Symptom frequency among patients receiving outpatient chemotherapy.	The most commonly reported symptom was fatigue. Fatigue is a common symptom in advanced-stage patients, but is usually undetectable if not questioned.
Karciga and Oflaz [178]	Turkish	Oral presentation	Cross-sectional	Quality of life (QoL) and anxiety levels of parents of children with metastatic cancers.	Cancer and its treatment (53.1%) and the inability to go to school (23.8%) were the most important problems affecting daily life of children. There was no difference between the age and education level of the parents and the child’s age, gender, disease duration, cancer type and metastatic organ and QoL scores. However, QoL of housewives (43.18 ± 15.53) and retired parents (42.45 ± 17.33) were higher than self-employed (33.78 ± 16.72) workers (38.75 ± 16.82) and civil servants (30.87 ± 15.15). The chronic disease of the parents negatively affects the total QoL and psychosocial health score (*p* < 0.05). The QoL scores of children who can go to school (44.67 ± 17.34) were higher than those who cannot go to school (37.16 ± 16.10), as well as those with a good financial status (44.24 ± 20.75) compared to other groups (*p* < 0.05).
Şener *et al* [246]	Turkish	Oral presentation	Cross-sectional	Care burden and life satisfaction of caregivers in the PC unit.	There was no difference between the mean scores of Life Satisfaction Scale and Care Burden Scale of the study and control group patients’ relatives. There was no correlation between the mean scores of the Life Satisfaction Scale and the Caregiving Burden Scale (*p* > 0.05).
Dokuyucu *et al* [72]	Turkish	Oral presentation	Cross-sectional	PC knowledge of the healthcare professionals.	It was found that the PC awareness levels of healthcare staff were moderate and there were no major differences between the doctors and nurses in terms of correct answers.
Dokuyucu *et al* [236]	Turkish	Oral presentation	Cross-sectional	Satisfaction of patients from nursing service in PC centre.	Patients usually treated in PC centres have more depression. Considering this, it was concluded that the care of the nurses makes a high level of satisfaction.
Kırsever *et al* [188]	Turkish	Oral presentation	Interventional	Palliative bioresonance therapy in metastatic cancer patients.	Significant symptomatic improvement and palliation were observed with bioresonance in advanced stage cancer patients.
Tapar *et al* [150]	Turkish	Oral presentation	Cross-sectional	Frequency of neuropathic pain in patients with colon cancer.	The analgesics most frequently used were oxycodone 2.7%, antidepressant 12.2%, haloperidol 21.6%, morphine tablet 25.7%, fentanyl transdermal 31.1%, tramadol tablet 44.6%, anticonvulsant (gabapentin, pregabalin) 54.1% and Nonsteroidal anti-inflammatory + paracetamol 60%. The 54.1% of patients using anticonvulsant medication are using an opioid medication together.
Erkal *et al* [192]	Turkish	Oral presentation	Cross-sectional	Evaluation of PC patients treated in the tertiary intensive care unit.	It was found that 21.4% of intensive care patients were cancer patients and need PC and the mortality was 70%. The vast majority of patients requiring PC are treated in intensive care units due to the lack of adequate beds in the services. This increases the cost and eliminates the possibility of treating patients who need intensive care but have the chance to survive.
Bozkurt M [299]	Turkish	Oral presentation	Retrospective/cross-sectional	Survival of advanced gastric adenocarcinoma patients referred to the supportive care unit.	It was found that 50% of patients with an expected life expectancy of ≤ 3 months lived for 1.5 months when referred to supportive treatment.
Çakır and Uzuner [300]	Turkish	Oral presentation	Cross-sectional	The comfort of children with cancer and their parents with deep sedation applied for invasive procedures in inpatient unit.	Invasive procedures in children with cancer can be performed safely with anaesthesiology guidance. Deep sedation in inpatient unit both prevents the pain of the patients and eliminates the concerns of the patients and parents.
Pilatin and Araç [142]	Turkish	Oral presentation	Retrospective	Retrospective analysis of patients hospitalised in the PC unit in Diyarbakır.	The average age of the 203 patients was 67 (20–95) years. It was found that 54.5% of the patients were hospitalised due to malignancy and complications related to malignancy. Average hospital stay was 10.8 days. About 42% of the patients were discharged, 13% were expired and 45% were referred to another centre.
Toprak and Pilatin [143]	Turkish	Oral presentation	Retrospective	Retrospective analysis of patients hospitalised in the PC unit.	A total of 137 hospitalisations of 98 patients were detected. The number of hospitalisations due to malignancy and malignancy-related complications was 108 (78.8%). Average hospital stay was 11.9 days. Forty (90.9%) of the patients who died were hospitalised due to malignancy.
Akelma and Platin [71]	Turkish	Oral presentation	Cross-sectional	Views of healthcare personnel working in a Training and Research Hospital regarding PC.	A total of 87 healthcare personnel (73 nurses, 3 doctors, 11 other) were included. 64.4% of the healthcare personnel stated that they did not receive formal education about PC, 54.8% learned PC in university, 91.3% stated that there was no training programme about PC in their institution. They think that patients who can benefit from PC the most were cancer patients (72.7%) and terminal period patients (54.5%). They think the purpose of PC is ‘relieving the pain and psychological relief of end-stage patients’. Almost all individuals participating in the study stated that nurses, doctors and psychologists should be included in the palliative team and also pharmacists, volunteers and religious officials might be included. 76.5% of them stated that PC can be provided in hospital. The reason of why PC services have not yet developed in our country was lack of knowledge and training about palliative units according to 91.2% of participants. The need for training programmes was basic concepts of PC (95.8%), communication and bereavement (77.7%). 80.9% of the healthcare personnel think that ‘Palliative care is for terminal stage cancer patients’, 85.7% think emotionally enhancing programmes should only cover the caregivers. 80.7% of them stated that they agree/totally agree with the statements ‘the employee should control his/her emotions’.
Eren *et al* [179]	Turkish	Oral presentation	Cross-sectional	The effect of support and evaluation of home care on hospitalisation frequency and quality of care in cancer patients requiring PC.	It shows that the lack of knowledge and experience of caregivers about terminal cancer patient care is an important factor for frequency and desire of hospitalisation. The terminal stage is an inevitable period in cancer. End of life care loads more already busy oncology clinics. In the later stages of our study, it was aimed to analyse the cost and develop a hospitalisation scale that combines the factors studied.
Topkaya *et al* [161]	Turkish	Oral presentation	Retrospective/cross-sectional	Symptoms among patients died with cancer.	Study included 45 cancer patients who were followed up in the oncology clinic and died. Palliative Performance Scale of all were 40% and below. Seventy-six percent of patients experienced more than one symptom and 7% had pain, insomnia, respiratory distress and constipation together. Other 82% had pain, 41% had pain & insomnia, 31% had constipation, 57% had respiratory distress, 64% patients had respiratory distress, 41% of them experienced insomnia and 72% of them experienced pain. 29% patients had constipation and 92%of them had also pain. Planning the appropriate treatment and care according to the complex symptoms increases the success in symptom management and increase the quality of life in end-of-life care.
Özyalçın and Çevik [250]	Turkish	Oral presentation	Cross-sectional	The attitudes of cancer patients, their caregivers and nurses towards death.	The mean score of the well-being scale and the attitude towards death scale (DAP-R) of the patients participating in the study was higher than the relatives and nurses, and the difference between them was found to be statistically significant. A significant relationship was found between the age, gender, marital status of the patients, and, the fear of death, death avoidance and acceptance.
Topkaya and Yürügen [214]	Turkish	Oral presentation	Cross-sectional	PC needs of cancer patients and to examine the knowledge and expectations of patients and their families.	The symptoms experienced by the patients according to the Edmonton Symptom Scale were fatigue 76.4%, feeling unwell 73.6%, loss of appetite 66.4%, pain 64.5%, worry 62.7%, insomnia 56.4% and nausea 55.5%. About 76.4% of the patients and 60% of the families stated that they had never heard of PC. While 25% of those who heard of PC defined PC as end-of-life care, others defined it as symptom management and supportive treatment with various expressions.
Altınışık *et al* [233]	Turkish	Oral presentation	Cross-sectional	Supportive care needs and hope levels of caregivers of cancer patients.	There was no significant difference in the sociodemographic characteristics of the patients and the unmet care needs of the patients and their caregivers. Significant differences were found in the stage of the disease, the duration of diagnosis, the treatment status in the last 2 months, the knowledge and the income level of the caregiver. The most needed healthcare service was pain control (39.3%). The average score of the hope levels of the caregivers is 35.52 ± 7.66. Regression analyses showed that the advanced stage of the disease increased the needs of the caregivers, and the treatment of the patient in the last two months decreased the health care needs.
Özsoy *et al* [151]	Turkish	Oral presentation	Cross-sectional	Use of surgical procedures among PC cancer patients.	A total of 75 patients underwent palliative surgery. The most common symptoms were abdominal pain, vomiting and weakness. Indications were; oral intake disorder, bleeding, ileus, perforation, bile drainage deterioration (jaundice). Stomach, colon and rectum were the most frequently affected organs.
Kuşçu *et al* [152]	Turkish	Oral presentation	Retrospective	Retrospective analysis of patients who underwent acid palliative treatment via catheter insertion.	One hundred and seventy-nine oncology patients were evaluated. Procedure reduces the number of repetitive paracentesis, provides symptomatic relief, increases the quality of life of the patient and his family. It also reduces the number of clinical visits. No infection was seen except for local infections due to catheter.
Kılıçkap *et al* [162]	Turkish	Oral presentation	Cross-sectional	The relationship between quality of life and clinicopathological features in cancer patients.	The data of 1,549 cancer patients were analysed. The most frequently observed diagnoses were breast cancer (21%), haematologic cancers (18%) and colorectal cancer (11%), respectively. Individuals with ECOG performance status ‘0’ had higher quality of life scores (QLS) (*p* ≤ 0.001). As the stage increased, the QoL decreased significantly (*p* ≤ 0.001). In patients with anaemia (10g/dL), hypoalbuminaemia (3.2 g/dL), and leukopenia (3,000/mm^3^), QoL was significantly lower (*p* ≤ 0.001). Inpatient treatment (*p* ≤ 0.001), comorbid disease (*p* ≤ 0.001), family history of cancer (*p* ≤ 0.001), active treatment (*p* ≤ 0.001), admitted within the first year after diagnosis (*p* ≤ 0.001) and patients who had relapse (*p* ≤ 0.001) had significantly lower QoL. Patients with distant metastases had lower QoL compared to patients with local metastases or non-metastases markedly (*p* ≤ 0.001). According to the presence of the disease and the status of receiving treatment, the general QoL of the patients was found to be significantly different (*p* ≤ 0.001). Patients without active disease and who did not receive any treatment, the overall QoL was the highest, while others who had terminal disease and received active treatment had the lowest (*p* ≤ 0.001). No relationship was found between gender, marital status, previous cancer history and age and overall quality of life score.
Aslan and Akyol [228]	English	Poster	Cross-sectional	Hope levels of family caregivers of cancer patients and demographics.	This research implied that family caregivers of cancer patients had sufficient level of hope. Nevertheless, hope levels of family caregivers can be reached to higher levels by supportive care activities.
Bagcivan *et al* [164]	English	Poster	Cross-sectional	Evaluation of the relationship between level of nursing care satisfaction and symptom experience due to chemotherapy in cancer patients.	The total mean score of satisfaction scale was 4.37 ± 0.81 showing high patient satisfaction. It is a positive result for nursing care. There were no significant difference between patient socio-demographic variables and satisfaction mean score. The most frequent symptoms of patients were lack of appetite (*n* = 50, 83.3%), debility (*n* = 49, 81.7%) and nausea (*n* = 41, 68.3%). There were no relationship between patient symptom severity and satisfaction mean score (*r* = −0.094, *p* = 0.474).
Özçelik *et al* [247]	English	Poster	Cross-sectional	The relationship between caregiving burden and social support in caregivers of patients with cancer.	There were no significant differences between mean caregiving burden, social support scores and some socio-demographic characteristics (marital, employ, occupational and to be primary caregiver status). The caregiving burden levels of the caregivers were lower in patients with higher educational background. There was a negative weak significant correlation between the mean caregiving burden scores and social support scores (*r* = −0.29, *p* = 0.000). The results of the study show that caregiving burden of caregivers can be reduced by increasing social support.
Okcin *et al* [237]	English	Poster	Cross-sectional	Nursing care satisfaction of patients receiving chemotherapy.	Patients are influenced by nurses’ personal behaviours and outpatient chemotherapy patients want sufficient information and emotional support.
Yetisen *et al* [229]	English	Poster	Cross-sectional	Scoring anxiety, depression and quality of life in colorectal cancer patients and the effect of patient education on these parameters.	There were not statistically significance changes in anxiety, depression and the quality of life scores between groups. Changes in the State-Trait-Anxiety-Inventory (STAI) scores were statistically significant in study group after education. The Short-Form-Health Survey (SF-36) measures eight domains of health. We compared each domain and physical functioning score was found statistically significant between groups.
Yavas *et al* [180]	English	Poster	Cross-sectional	Quality of life in patients with malign glioma: a prospective study in Turkish Population.	There were many changes about parameters related to both quality of life and cognitive functions compared to baseline scores and follow-up scores. Most of them were found to be related to disease progression. We did not observe any depression and anxiety in our patients.
Yavas *et al* [301]	English	Poster	Validation of scale	The assessment of Health Related Quality of Life (HRQOL) in adult patients treated for low-grade glioma.	To assess quality of life, cognitive and emotional distress on patients with diagnosis of high-grade glioma. European Organization for Research and Treatment of Cancer Quality of Life Questionnaire 30 (EORTC-C30), Brain Cancer Module-20 (BN-20), Mini Mental Standard Examination (MMSE) and Hospital Anxiety and Depression Scale (HADS) were administered. There were a statistically significant difference regarding to cognitive function scores of the patients who used or did not use antiepileptic drugs (*p* < 0.001). We did not observe any depression and anxiety in our patients.
Erol *et al* [165]	English	Poster	Cross-sectional	Pain experiences of patients with cancer: a phenomenological Study.	Patients with pain experienced fear and anxiety, restrictions in daily life and constrained in pain management. The main themes that emerged were pain perception, restrictions in daily living, pain management and coping. The results of this study can increase nurses’ awareness of their role in pain management. Patients need much more attention of health professionals for pain control.
Koc *et al* [166]	English	Poster	Cross-sectional	Fatigue level and affecting factors in oncologic patients.	Piper Fatigue Scale and Brief Fatigue Inventory were used. The patients who were operated on with the diagnosis of cancer suffered from moderate and intense fatigue levels, while the patients in the control group experienced a mild level of fatigue.
Yildiz *et al* [187]	English	Poster	Interventional	The effect of individual physiotherapy and rehabilitation training programme on lymphedema in patients with mastectomy.	Patient-specific home training programme may decrease the amount of oedema and pain. The education given to patients with breast cancer and candidates of lymphedema during the early stage may increase the success of the treatment.
Çıracı and Nural [230]	English	Poster	Cross-sectional	Loneliness in cancer patients.	Oncology patients at end of their life felt high rates of loneliness.
Tanriverdi *et al* [302]	English	Poster	Cross-sectional	The perspective of non-oncologist physicians on metastatic cancer patients and PC (ALONE study): Palliative Care Working Committee of the Turkish Oncology Group.	A total of 71% of participants identified all metastatic patients as being terminal-stage, 62% were unaware of PC techniques, 64% did not know about common supportive care options, 59% were against hospice and 63% had no opinion on resuscitation.
Çıracı and Nural [231]	English	Poster	Cross-sectional	Emotional status of cancer patients at end of life.	Cancer patients not only feel physical symptoms but also feel negative emotions such as loneliness, bargaining, not being able to be with close friends at the end of life and they need psychological support.
Koc *et al* [23^2]^	English	Poster	Cross-sectional	Loneliness in oncologic patients and social support levels.	The patients had lower perceived social support levels and loneliness levels compared to the control group.
Sahin *et al* [67]	English	Poster	Qualitative	Thoughts of nursing students about PC.	Nursing students find their curriculum is superficial for PC and would like it to be strengthened. They suggest that this specialty content be expanded as part of a speciality degree programme.
Cetintas *et al* [68]	English	Poster	Cross-sectional	Death anxiety and attitudes toward the principles of dying with dignity of nursing students.	Death Anxiety Scale (DAS) and Assessment Scale of Attitudes Toward Principles About Dying with Dignity scales were used. There was a significant difference between the students who witnessed the death of a child on ‘uncertainty of death’ subscale mean scores of DAS (*p* < 0.05) and Assessment Scale of Attitudes Toward Principles About Dying with Dignity scores (*p* < 0.05). There was a negative correlation between the number of deaths that students witnessed and total scores of DAS (*p* = 0.045).
Seven *et al* [167]	English	Poster	Cross-sectional	PC needs of patients with cancer.	The adapted Edmonton Symptom Assessment System, Beck Depression Inventory (BDI) and Beck Anxiety Inventory (BAI) were used to collect data. Some symptoms are experienced by most of the patients and some symptoms are felt by fewer patients, but more severely. To reduce the negative effects of both physical and psychological symptoms associated with cancer and its treatment, nurses should evaluate these symptoms. The implementation and assessment of the nursing attempts should be tailored for each individual.
Özkan [303]	English	poster	Review	Self-care and end-of-life care for cancer patients and caregivers.	There are important limitations of cancer self-care and PC studies in assessing the cultural differences and failure to cover all of the outcome measures. Researchers need to build self-care and PC that paralleled advances in clinical research and practice intended for cancer patients.
Koc *et al* [181]	English	Poster	Qualitative	Life quality, satisfaction and pain perception in cancer patients.	Patients evaluate their life quality as a whole, that their general wellness is very low, that they are partially not happy with their life satisfaction and that they have more than average pain perception.
Çolak *et al* [129]	English	Poster	Cross-sectional	The attitudes of cancer patients about morphine usage for pain management.	The age (*p* = 0.010) and gender of the patient (*p* = 0.038) and their knowledge about morphine (*p* = 0.000) had a statistically significant effect on the preference of the patients. The patients who defined morphine as narcotic drug were less likely to use morphine (*p* = 0.015). There was no relationship between the patients’ preference and the diagnosis (*p* = 0.247), the stage (*p* = 0.552), the education status (*p* = 0.112) and the pain. The risk of addiction to opioids remains a major obstacle preventing effective pain management.
Cakir and Kaygusuz [304]	English	Poster	Cross-sectional	Nasogastric tube feeding in children with cancer as a part of PC.	Weight loss is an important problem in patients with cancer. Patients who lost weight should be fed by nasogastric tube (NG). Palliative enteral feeding by NG tube is safe, inexpensive, and has a low complication rate. NG feeding, rather than PEG, could be better than enteral feeding in children with cancer.
Bağçivan *et al* [305]	English	Poster	Validity and reliability of scale	Analysis of the patient-related barriers in cancer pain management in Turkish patients.	Barriers Questionnaire-II (BQ-II) was a valid and reliable scale for patient related barriers in cancer pain management. Items suggested by patients such as ‘family related barriers’ and ‘family members’ role and responsibilities’ could be added to the BQ-II for future studies.
Eskigülek and Kav [234]	English	Poster	Validity and reliability of scale	Turkish validity and reliability of the Patient Dignity Inventory (PDI) among PC patients and to explore the views of PC patients and nurses about dignified care.	Cronbach’s coefficient alpha for The Patient Dignity Inventory (PDI) was 0.94 and test–retest reliability was *r* = 0.75. Concurrent validity tests demonstrated positive significant correlations between factors of PDI and Hospital Anxiety and Depression Scale (HADS). Factor analysis demonstrated five factors accounting for 68.7% of the overall variance. The factors were labelled as symptom distress, existential distress, self-confidence, dependency and, support and care requirements. Three themes emerged through data obtained from PC patients: respectability, caring practices and usefulness. Three themes emerged through data obtained from PC nurses: maintaining one’s respectability, barriers and recommendations and benefits of care.
Gok Metin *et al* [168]	English	Poster	Randomised controlled trial	The effect of mindfulness-based stress reduction (MBSR) and progressive muscle relaxation (PMR) for breast cancer patients receiving adjuvant paclitaxel regimen.	Brief Fatigue Inventory (BFI) scores were significantly decreased in the MBSR, PMR groups compared with the control group. The sub-dimension scores of Brief COPE including denial, behavioural disengagement, acceptance, humour, using emotional support, using instrumental support, substance use, planning and positive reframing were significantly higher in the MBSR and PMR groups. Regarding FLIC scores, there were no significant differences between the groups.
Kılıçkap *et al* [163]	Turkish	Poster	Cross-sectional	Impact of the quality of life at the time of diagnosis on overall survival of cancer patients.	Data of 457 patients were analysed. The most common diagnoses were lung (19%), haematologic (14%) and colorectal (10.5%) cancer, respectively, and 51% had stage 4 disease. ECOG PS were zero in 60% of the patients. Sixty-eight percent of the cases were on treatment: 87% were receiving chemotherapy and 29% were treated inpatient. Comorbid diseases were found in 26 percent of the cases. Advanced stage, low haemoglobin and albumin level, ECOG PS ≥ 1, hospitalisation, comorbid disease and distant metastasis were associated with quality of life (QoL) score. The median follow-up period was 25 months (1–60). Overall survival was significantly lower in patients with a low QoL score at the time of diagnosis (median 10 versus 40 months; *p* ≤ 0.001). The 3-year OS was lower in individuals with low QoL (27% versus 52%). In multivariate analysis, stage (*p* ≤ 0.001), hypoalbuminaemia (*p* ≤ 0.001), ECOG performance score (*p* ≤ 0.001), and low QoL (*p* = 0.022) were independent variables affecting survival. Low quality of life score at diagnosis is an independent variable affecting survival. Application of the QoL scale may be useful in every newly diagnosed patient.
Sezer *et al* [145]	Turkish	Poster	Retrospective	Characteristics of patients who received inpatient treatment in PC centre between 2008 and 2009.	During a year 352 patients were hospitalised in 557 times. Their gender was 55% were male and 45% were female, and the mean ages for both sexes were 56.3 and 54.5. Diagnoses were lung cancer (27.8%), gastrointestinal system cancer (20.2%), haematological malignancy (9.9%), breast cancer (10.8%), gynaecological cancer (8.8%), head and neck tumours (7.6%), urogenital (5.4%), malignant melanoma (2.4%), nervous system tumour (2.8%), soft tissue and bone tumours (4.6%). Reasons for hospitalisation were: diagnosis and chemotherapy (35.7%), complications (34.7%) and PC (29.6%). Average hospital stay was 6 days for diagnosis and chemotherapy, 9 days for complications and 18 days for PC. Major complications were infection and bone marrow depression (42%). Most of the patients hospitalised for palliative purposes were for pain palliation and nutrition support. Compared to the previous year, it was found that the duration of hospitalisation for PC was prolonged, and the duration of diagnosis and chemotherapy was shortened. Our aim is to increase PC opportunities, to shorten the complication rates and the duration of hospital stay by making good follow-up after chemotherapy.
Kömürcü *et al* [197]	Turkish	Poster	Cross-sectional	Tests and treatments performed during the last 2 weeks among terminal stage cancer patients who died.	There were 422 metastatic cancer patients with a life expectancy of 6 months. Causes of death in order of frequency; respiratory failure (48.1%), infection (23.5%) and/or liver failure (18.9%). The examinations performed during last 2 weeks were; 22.7% computed tomography, 23.2% ultrasonography, 13.7% magnetic resonance imaging and 4.9% bone scintigraphy. In all 76.9% of the patients received intravenous serum treatment, 43.6% erythrocyte transfusion, 36.5% total parenteral nutrition and 6.9% albumin transfusion. It was observed that 25.7% received invasive pain treatment, 12.4% terminal sedation with morphine or midazolam and 9.1% chemotherapy. In addition, vascular access was established in all patients, a central catheter and/or urinary catheter was inserted in 37.9%; It was found that 10% had paracentesis, 7.3% thoracentesis and 2.9% endoscopy.
Doruk e*t al* [201]	Turkish	Poster	Cross-sectional	Pain management and supportive treatments in terminal cancer patients in hospital.	The average length of stay in the hospital was 16.9 (2–74) days. Chemotherapy (CT) was given in 44% of the patients and 45% of them received CT within 14 days before death. The average time from CT to death was 59.9 (3–138) days. During their hospitalisation, invasive interventions (42%), parenteral nutrition (16%), albumin infusion (50%) and fresh frozen plasma (12%) were given. A significant relief was noted for symptoms of pain, anorexia, nausea, vomiting, constipation, shortness of breath and insomnia with the supportive treatment (*p* = 0.002 - *p* = 0.008).
Tanrıverdi and Yıldırım [306]	Turkish	Poster	Retrospective/cross-sectional	Evaluation of end-stage cancer patients who applied to the emergency department 1 month before their death.	Study group consisted of 107 deceased cancer patients who applied to the emergency service. In Group 1, there were 64 patients admitted to the emergency department at least once in a month before their death. Remaining 43 had never needed the emergency department within 1 month before their death (Group 2). Thirty-eight percent of patients in group 1 had lung cancer and the most common reason for presentation was dyspnoea (92%) and 26% of the patients in group 2 had colorectal cancer. In Group 1, 38% of the patients were discharged from the emergency room and 9% died in the emergency room. It was found that 10% of the patients in group 1 were hospitalised in ICU, and the rest were hospitalised in: oncology (28%), chest or infectious diseases (11%) and general surgery (4%) wards. Patients who applied to the emergency department. In the last month of their lives were positively correlated with tumour location (primary lung or lung metastasis), good performance status, presence of pleural effusion and the presence of pain.
Ekinci *et al* [144]	Turkish	Poster	Cross-sectional	Characteristics of patients treated in a PC centre.	Most patient had cancer. Among the reasons of admission, the first was nutritional support.
Özsoy [267]	Turkish	Poster	Review	Biometric analysis of PC publications in the period 2000–2016.	It was observed that the number of publications in the field of PC increased significantly after 2006. The USA and the UK are pioneers in the number of publications.
Mutafoğlu *et al* [51]	Turkish	Poster	Cross-sectional	Attitude of oncologists about PC in cancer.	Almost 97% of the participants find the PC services provided to patients with cancer in our country are inadequate. Despite supportive treatments for physical symptoms, psychosocial support is insufficient.
Topaloğlu *et al* [149]	Turkish	Poster	Cross-sectional	Factors affecting pain control in patients with cancer pain.	About 58% of the patients had pain for more than 2 months. 64% of the patients with poor pain control did not use regular analgesics. The most common reason for not using regular analgesics was ‘not being recommended that analgesics should be used regularly’.
Çakır and Uzuner [160]	Turkish	Poster	Case series	Nutritional support result of children with nasogastric tube.	In case of weight loss among children with cancer, if nutrition cannot be provided orally, it should be provided via the nasogastric (NG) route. NG feeding is a safe, cheap, and uncomplicated nutritional care.
Tosun *et al* [183]	Turkish	Poster	Cross-sectional	Nutritional support of patients with lung cancer receiving PC: difficulties experienced by patients and caregivers.	Caregivers are anxious about the nutrition of their patients and they thought that alternative nutrition methods were not sufficient. Bed dependence, nausea, vomiting, anorexia, cachexia, pain, shortness of breath and personality changes were the most frequent symptoms. Family structure and cultural differences may affect individual differences in nutrition-related patients. Nutritional support should be added to planning of care of patients with cancer.
Yıldız Savran *et al* [154]	Turkish	Poster	Cross-sectional	Symptom clusters among children with cancer.	Age, time to diagnosis and number of chemotherapy cycles affect the symptoms of the children. The number of symptoms increased with higher age, longer time to diagnosis and higher number of chemotherapy cycles. Children who had their treatment in the hospital experienced physical symptoms more frequently. The most frequent symptoms were low appetite, irritability and difficulty in concentration.
Söğütlü Çetin and Kuran Akburak [155]	Turkish	Poster	Cross-sectional	Symptoms experienced by patients receiving chemotherapy and factors affecting these symptoms.	The symptoms of fatigue and anxiety were found to be significantly higher among inpatients. Whereas constipation, alopecia, mouth problems were more frequent among outpatients.
Akyüz [211]	Turkish	Poster	Cross-sectional	PC practices for cancer patients.	Satisfaction level of cancer patients treated in hospitals with PCU was higher than others without PCU. Patients receiving treatment in the PCU do not want to go another hospital, and they want to receive psychological support as well as medical support during the treatment.
Kuşçu *et al* [249]	Turkish	Poster	Prospective/cross-sectional	Benefits of Palliative Performance Scale (PPS) for planning end-of-life care for patients.	Palliative Performance Scale can be used as a powerful tool in predicting prognosis and survival together with clinical and laboratory findings.
Alkan *et al* [50]	Turkish	Poster	Cross-sectional	Opinions and competency of internal medicine residents on pain management in cancer patients.	Pain management for cancer patients is an important issue that internal medicine residents should learn in the training process. The rotation period should be appropriate with the relevant education and medical experience.
Demir and Yılmaz [307]	Turkish	Poster	Cross-sectional	Expectations of oncology patients and their relatives from home care services.	Oncology patients do not benefit from home care services at an adequate and desired level, and home care services are yet to be effective and available at meeting the expectations of oncology patients.
Dayanç *et al* [147]	Turkish	Poster	Cross-sectional	Analysis of self-symptoms and problems of patients discharged after chemotherapy and counseled by telephone.	The most common problem for consultation were nausea (29.1%), vomiting (19.5%), diarrhoea (14.5%), pain (10.8%), constipation (9.16%) and loss of appetite (4.16%).
Abca Yılmazer and Okyayüz [227]	Turkish	Poster	Interventional	Information-focused pain and depression assessment in patients with bone metastasis during palliative radiotherapy.	Patients with bone metastasis were divided into two groups. Both were offered appropriate analgesic treatment and palliative radiotherapy to painful bone metastases. Psychological pain control methods (relaxation, breathing exercises and cognitive therapy) were recommended to interventional group. Semi-structured interview form were applied and the visual analogue scale for pain assessment and Beck Depression Scale for the evaluation of depression were used for comparing interventional and control groups. Post-treatment Beck Depression Scale levels were found to be higher in the interventional group than in the control group. We recommended to use psychological methods in addition to pharmacological methods.
Özçelik *et al* [158]	Turkish	Poster	Cross-sectional	Determination of family satisfaction levels of advanced cancer patients.	The family satisfaction total scale mean score was 10.7676 ± 13.28. Other subscale mean scores were as follows: getting information as 10.03 ± 13.85, access to care as 11.20 ± 14.82, physical care as 10.64 ± 14.21, psychosocial care as 11.46 ± 14.30.
Demirkaya *et al* [159]	Turkish	Poster	Cross-sectional	Evaluation of pain experiences by paediatric oncology patients.	The pain complaint rates are high in paediatric oncology practice. The quality of life and treatment compliance of children with cancer is affected by many factors, mostly due to pain.
Görgün *et al* [130]	Turkish	Poster	Cross-sectional	Pain assessment and palliation in patients with metastatic cancer.	It was aimed to explore pain status of patients and propose an algorithm for treatment approach in patients with advanced stage cancer who were followed up in hospital, and to emphasise the importance of questioning the patient’s pain. In addition, this importance of the pain, even in inpatients, reveals the necessity of careful pain management in outpatient follow-up. Pain palliation, which is one of the cornerstones of palliative treatment, should always be kept in mind in the treatment of cancer patients.
Urlu *et al* [131]	Turkish	Poster	Cross-sectional	Interventional pain management among oncology patients.	The vast majority of cancer patients can be treated with appropriate medical treatment for pain. Interventional methods should be tried in patients who do not respond to medical treatment. For this, palliative treatment teams should work with a multidisciplinary approach.
Aktaş *et al* [308]	Turkish	Poster	Case report	Nursing care for a patient with cholangiocarcinoma.	PC was planned with a multidisciplinary team in hospital and coordinated support continued at home. During inpatient stay ascites palliation, pain control, nutrition and antiemetics were planned and his performance and quality of life increased.
Tiryaki *et al* [309]	Turkish	Poster	Case report	The role of the nurse in PC in a patient with lung cancer.	Patients and their relatives were trained on the management of nausea and vomiting and sleep issues and oral health. The onset, location, type, of pain, were determined. Analgesics were applied. The patient’s quality of life was improved by monitoring the pain during the treatment.
Demiral [310]	Turkish	Poster	Case report	Palliative nursing care for a patient with desmoplastic small round cell tumour.	There have been attempts to relieve pain, prevent infection, provide nutrition, prevent constipation, relieve anxiety and fears, and fatigue. The PC has improved patient quality of life.
Üçgül Çavuşoğlu *et al* [200]	Turkish	Poster	Cross-sectional	The treatments and tests performed in the last 2 weeks for patients with terminal cancer before death.	Infections and liver failure were leading cause of death in the last 2 weeks before death. Radiological examinations, total parenteral nutrition, albumin transfusion, enteral feeding, blood transfusion, pain relief and terminal sedation were noteworthy procedures found during this period.
Doruk *et al* [215]	Turkish	Poster	Cross-sectional	Opinions of terminal cancer patients and their caregivers about medical approaches and treatments applied in the hospital.	Generally, people other than the patient make the decision for hospitalisation in the terminal period. Informing patients and their relatives about terminal period care and their participation in decisions is important. An empathic and relevant approach is expected from healthcare staff. Psychosocial support is necessary for both the patient and their relatives.
Koyuncu and Aksu [132]	Turkish	Poster	Interventional	The effect of morphine sulphate infusion on pain control in terminal cancer patients.	The median visual analogue scale pain levels of the patients were found to be 8.5 (7–10) before morphine sulphate administration and 6.5 (4–7) after morphine sulphate administration.
Köse F [251]	Turkish	Poster	Cross-sectional	The indications and complications of terminal sedation with morphine and midazolam in emergency and elective conditions.	The aetiology for the refractory symptoms was dyspnoea, delirium and intractable pain for patients in elective and immediate sedation groups as follows: 51% versus 47%, 10% versus 19% and 38% versus 33%. Thorax region served primary site for the significant percentage of the patients with rates of 61% and 57% in elective and immediate sedation groups. The number of patients with intractable symptom of the delirium in immediate group (19%) notably higher than elective group (9%). Time between last chemotherapy and start of the palliative sedation was 71 versus 97 days in elective and immediate group, respectively.
Göksel and Doğan [140]	Turkish	Poster	Retrospective /cross-sectional	Home Health Services in Turkey (HHS) for gastrointestinal malignancies.	After neurological and psychiatric diseases, cardiovascular diseases and orthopaedic diseases, cancer patients constitute the fourth group disease that receives HHS most frequently. HHS started with a total of 593 teams in 2011 and increased to 662 teams in 2017. Total number of visits made in 2011 was 344,014, and visit number increased to 10,917,965 in 2017. 7,278 visits were made for patients with cancer in 2011, 74,261 in 2017. The total number of patient visits with GIS malignancy was 89,389 during 7 years.
Almaca *et al* [184]	Turkish	Poster	Case control	Effects of enteral nutrition (EN) and parenteral nutrition (PN) on clinical outcomes of cancer patients.	There was no difference between EN and PN in terms of overall mortality. Considering the patients who need intensive care during their hospitalisation; the rate of PN was significantly higher than EN. Thrombosis rates were similar between the two groups, but infectious complications were higher in the PN group.
Arias-Casais *et al* [311]	English	Book	Mixed-method; systematic review, surveys, interviews	To provide an updated analysis on the development and integration of PC across Europe.	The funds are allocated for PC from the Turkish health budget. PC is included in the list of health services provided at the primary care level and in the basic package of health services. The PC services in Turkey are 0.2 per 100,000 ınhabıtants. None of the medical and nursing schools offering specific mandatory PC courses in Turkey. Opioid consumption per capita in morphine equivalent in 2017 was 1.3 mg. PC resources for children were very limited.
Kömürcü and Türkiye’de Palyatif Bakım [17]	Turkish	Book	Review	This chapter provide overview of PC for cancer in Turkey during 2009.	As in many developing countries, the current status of PC in our country is not yet at the desired level. The main problems for the development are lack of adequately trained personnel, Opiophobia in the public and healthcare workers, lack of laws and regulations regarding PC, and insufficient financial support.
Utku *et al* [44]	English	Book chapter	Review	This chapter gives the narratives of Turkey’s experience, challenges and successes for the PC efforts.	After the 5 years of the Pallia-Turk Project, Turkey has 226 PC services, 947 home-care teams, 21,696 family physicians and nurses serving for primary level care. Oral morphine tablets with different dosage formulas have been produced by 2015. The opioid accessibility and usage have increased due to reimbursement by the Social Security Institutes in the country within the last years. Legislation regarding the integration of PC services at the primary level, integration of family physicians to home care teams, patients’ rights for rejection treatments, reimbursement of PC services in-home care and in the hospital were published.
Temelli [312]	Turkish	Thesis	Cross-sectional	To identify the perceptions of palliative nurses about death and determining PC practices.	Palliative nurses perceive death as a natural and inevitable process and that as long as their working period increases, they become desensitised.
Topkaya [313]	Turkish	Thesis	Cross-sectional	To determine the PC needs of cancer patients and to examine the knowledge and expectations of patients and their families about PC.	Seventy-six point four percent of the patients and 60% of their families were not aware of palliative/supportive care.
Yılmaz [314]	Turkish	Thesis	Cross-sectional	To determine the frequency of symptomatic manifestations in cancer patients treated in Palliative Care Unit and examine changes in the frequency after PC.	A statistically significant difference was found between admission and discharge values in terms of the severity of pain, fatigue, nausea, sadness, anxiety, insomnia, anorexia, shortness of breath and paresthesia in hands.
Arkın [315]	Turkish	Thesis	Cross-sectional	To evaluate the psychosocial features of family caregivers of PC patients at an inpatient clinic by examining perceived social support, depression and anxiety.	The increase in social support perceptions of caregivers of PC patients was associated with a decrease in the symptoms of anxiety and depression.
Sağlam [316]	Turkish	Thesis	Methodological	To determine the needs of family members who care for palliative patients regarding the care and treatment processes.	The needs of the caregivers in relation to care and treatment processes differed according to their socio-demographic and care burden characteristics. Family Needs Scale is a valid and reliable measurement instrument that can be applied to palliative patients’ relatives in our country.
Erdem [205]	Turkish	Thesis	Prospective cohort	To monitor individuals diagnosed with life-threatening diseases in a comprehensive PC centre run by family physicians and to evaluate their quality of life and related factors with early PC.	All subscale mean scores of quality of life assessment were higher in the 6th month of the study. While there was a significant positive correlation between the time from diagnosis to PC application and the quality of life in the energy and pain sub-dimensions, no significant correlation was found between the time from diagnosis to PC application and survival. Patients should be informed at the earliest stage.
Taş [317]	Turkish	Thesis	Prospective cohort	To investigate the effects of nutritional patterns on activity and performance scores, symptom levels, laboratory parameters, anthropometric measurements and mortality in PC patients with a high malnutrition risk score.	Among PC patients with malnutrition the patients who have lower activity and performance scores need more parenteral nutrition, and parenteral nutrition need, edema and poor performance status are independent determinants of mortality.
Düzgün [91]	Turkish	Thesis	Interventional	To investigate the effect of music on pain, anxiety comfort and functional capacity of cancer patients received care in a PC unit.	Turkish classical music therapy improved pain, anxiety, comfort, functional capacity, and the control of vital signs in cancer patients cared in the PC unit.
Tanıl [318]	Turkish	Thesis	Cross-sectional	To evaluate the opinions of the oncology patients in the palliative period about their experiences of bad news interviews and communication preferences for receiving bad news in addition to death anxiety and attitudes towards death.	The perceptions, expectations and attitudes of the palliative oncology patients on bad news were evaluated.
Ardıç [319]	Turkish	Thesis	Cross-sectional	To investigate if there is a significant relation between palliative stage cancer relatives’ self-compassion levels and psychological resilience, quality of life, depression, anxiety and stress.	Receiving information support had a significant effect on psychological resilience and receiving sufficient support had a significant effect on quality of life.
Duman [320]	Turkish	Thesis	Cross-sectional	To determine the effect of the opinions of the relatives of the inpatients in the Palliative Care Unit on the caregiver burden.	The opinions of the relatives of the patients about PC did not significantly affect the caregiver burden. However, caregiver burden was significantly affected according to the patient’s view of being peaceful in the hospital.
Damak [321]	Turkish	Thesis	Cross-sectional	To determine the nurses’ perceptions of good death and their knowledge about PC.	It was determined that the nurses’ knowledge of PC was moderate and their perceptions of good death were high.
Karakaya [322]	Turkish	Thesis	Cross-sectional	To evaluate the quality of life of caregivers (family members) of PC patients and to determine the factors affecting the quality of life of caregivers.	Quality of life levels of those with high age, those with low education level, those with low income and those with children were lower.
Kurtgöz [323]	Turkish	Thesis	Interventional	To determine the effect of nursing care on spiritual well-being and hopefulness in relatives of PC patients.	The provision of nursing care based on the theory of Watson’s Human Caring decreased the severity of hopelessness in the patient relatives; however, it was found that it did change their spiritual well-being levels.
Tarakçı [324]	Turkish	Thesis	Cross-sectional	To determine the factors affecting the long-term hospitalisation and discharge status of the patients hospitalised in a PC service of a tertiary hospital for a period of 1 year, to determine preventable and curable conditions, to prevent the morbidity and mortality caused by unnecessary health expenditures and long hospitalisation.	Majority of patients who were admitted to the palliative service were cancer patients. Factors that increase the duration of hospitalisation were; the presence of infection, polypharmacy and pressure sores, and the use of antibiotics and analgesics. Factors related to discharge status were; infection status (*p* < 0.01), antibiotic use status (*p*:0.02), diet (*p* < 0.01) and opioid use (*p* < 0.01).
Demirgil [82]	Turkish	Thesis	Cross-sectional	To examine the knowledge levels of emergency service employees concerning PC, as well as their educational background and approach to end-of-life care and to investigate whether that has a correlation with ethical sensitivity or not.	Majority of the participants (63%) had not received PC training before. While the PC score average was found to be 49.10 ± 8.25; the total score average of the Ethical Sensitivity Scale was found to be 78.73 ± 20.97.
Çelik [325]	Turkish	Thesis	Interventional	To investigate the effects of bright white light application on fatigue and sleep quality in cancer patients receiving PC.	The application of bright white light reduces the fatigue levels and increases sleep quality and sleep duration of cancer patients receiving PC. There was no positive or negative late effects of light on fatigue level and sleep quality in the period following bright white light application.
AÇalışkan [209]	Turkish	Thesis	Cost analysis	To conduct a cost analysis of PC as a new service branch in healthcare field.	Total cost of the Palliative Care Center was TRY 1.034.235,26. 36% of the total cost consisted of raw material and supply costs, 33% staff costs, 20% external benefits and services, 7% pubic shares and 3% depreciation and wear and tear allowance. The centre’s income could not meet its expenses, and it lost TRY 158.235,26 in total. As income was calculated as same-day treatment, the centre lost TRY 54,19 per day.
Akbulut Şahin [256]	Turkish	Thesis	Qualitative	To investigate the level of benefiting from religious coping strategies by the relatives of cancer patient.	It was determined that the relatives of the patients used religious coping strategies widely and thought that it was advantageous to benefit from religious coping strategies. Benefiting from religious coping strategies in the treatment process might produce positive results for relatives of patients.
Özhan [326]	Turkish	Thesis	Cross-sectional	To measure the depression levels, perceived social support and the quality of life of relatives of terminal period patients.	Economical conditions get worse as the points of the quality of life decrease. The correlation between the educational status and quality of life points is positive.
Adanır [327]	Turkish	Thesis	Cross-sectional	To determine the compassion fatigue and coping styles with stress of nurses working on oncology and PC patients.	As the compassion fatigue of nurses increased, compassion satisfaction decreased but burnout increased.
Balcıoğlu [328]	Turkish	Thesis	Cross-sectional	To evaluate the nutritional status of 65 years and older patients followed-up in the PC unit.	Malnutrition is highly prevalent in patients with the need for PC.
Gürel Yavuzdemir [210]	Turkish	Thesis	Cross-sectional	To examine PC services in a PC centre.	Families’ satisfaction with pain management was effective in choosing post-discharge care place and families wanted to choose a PC centre. 81.0% of the families had no awareness of PC and 19.0% of them had awareness. In research, direct cost of illness was examined. The average unit cost was 22.128 TL. The unit cost of a day in hospital was found as 391 TL.
Altay [329]	Turkish	Thesis	Quasi-experimental	To determine the effect of PC training on the knowledge level of nurses about PC for nurses working in oncology wards and nurses who care for cancer patients.	The PC training effectively improved the knowledge level of nurses.
Uslu Şahan [330]	Turkish	Thesis	Mixed method; RCT & qualitative	To determine the effect of interprofessional simulation training on PC competencies to gynaecologic oncology, interdisciplinary education perceptions and teamwork attitudes of the students and to determine opinions, thoughts and suggestions of students about the use of simulation in interprofessional gynaecologic oncology PC training.	Training programmes which are used together with high fidelity simulation and hybrid simulations applications in interdisciplinary training should be integrated into the undergraduate education curriculums of the future cooperating health professions.
Kaya [331]	Turkish	Thesis	Cross-sectional	To renew and reorganise physicians’ training schedules according to any consultation required by a physician in Palliative Care Service.	The average of consultations for a patient was 5.48 ± 7.29 and the maximum number of consultations was 50; 19.9% of patients did not require any consultation, 12.1% had one consultation, 14.2% had two consultations. In 84.3% of the cases, the reasons for consultation were for diagnostic and treatment purposes and in 6.54% for preoperative interviews.
Metin [204]	Turkish	Thesis	Cross-sectional	To investigate the relationship between the health literacy level of PC service patient companions and patient care results and survival status of the patients.	Health literacy of the companions of patients receiving PC is associated with bedsore occurrence and survival.
Eskigülek [332]	Turkish	Thesis	Methodological	To investigate the views of the PC patients and the nurses working in this field about dignified care and to evaluate Turkish validity and reliability of the Patient Dignity Inventory.	It was determined that the Patient Dignity Inventory is a valid and reliable scale for Turkish society.
Güçlü [333]	Turkish	Thesis	Cross-sectional	To investigate the effects of hand grip strength and nutritional status on mortality in palliative patients in our region.	Malnutrition rate was determined as 82.90% for the patients treated in PC units.
Bağçivan [334]	Turkish	Thesis	Methodological	To analyse the validity and reliability of the Turkish version of the Functional Assessment of Chronic Illness Therapy-Palliative Care (FACIT-Pal) Scale.	Turkish version of the Functional Assessment of Chronic Illness Therapy-Palliative Care (FACIT-Pal) Scale is a reliable scale and can be used to asses and monitor the PC-specific quality of life in cancer patients.
İlhan [335]	Turkish	Thesis	Cross-sectional	To determine the effect of burnout on the social lives of relatives or carers who provide continuous care service as primary to PC patients.	As a result of our cultural values, we are able to reduce mental/psychological health problems the most. It may be possible to anticipate that the level of burnout and the standard of living will improve as a result of social support from the environment of cancer patients.
Öksüzoğlu [336]	Turkish	Thesis	Cross-sectional	To evaluate the relationship between depression-burnout level and maintenance period observed in caregivers of PC patients.	The rate of depression is higher in caregivers who care for parents or partner. It was understood that the personal success scores of the depressive cases were lower, extension of the maintenance period and the inability to obtain support from the family members facilitated the emergence of the burnout situation.
Karadeniz [337]	Turkish	Thesis	Cross-sectional	To investigate the effect of the PC service provided in a well-equipped PC centre on the management of different symptoms and personal performance levels.	PC patients, who are experiencing many symptoms simultaneously, are found to report only the most discomforting symptom. PC units, approached the patient in a holistic manner, and by providing symptom control, increased the performance levels.
Kahraman [338]	Turkish	Thesis	Cross-sectional	To determine the factors affecting the social support perception of the relatives of the PC and oncology patients receiving treatment in hospital services, to identify whether there is difference in social support perception between two services, to enlighten their perceptions of social support, contributing to the filling of the lack of information and literature on social work in the organisation of PC which is very new in Turkey and to draw attention to the problems of patient relatives.	Reassessment of PC services, the need to deal with not only patients but also their relatives, adaptation process to illness, identical with the death, support during grief period necessities in Turkey are mentioned.
Kara [203]	Turkish	Thesis	Cross-sectional	To determine the opinions of terminal cancer patients about PC.	Eighty-seven point eight percent of the participants have never heard of PC concept. Although most of the participants (62.4%) would like to look after their patients at home, they prefer hospital care (79%).
Kado [339]	Turkish	Thesis	Cross-sectional	To identify the problems faced by patients and their relatives who need PC and to offer solutions	The patient and the relatives of the patient faced various troubles in the PC process such as communication problems with physicians, nurses and health personnel.
Güney [340]	Turkish	Thesis	Cross-sectional	To contribute to the integration process of PC into Emergency Department (ED) by examining the needs and symptom severities with Speed, Edmonton and Karnofsky scales by using a new screening method of patients who applied to ED and who need PC.	PC is a maintenance that should not be ignored in the current health system. It is not possible for emergency services alone to undertake such care, but it is not possible to exclude emergency services from this care.
Filiz [341]	Turkish	Thesis	Cross-sectional	To determine the satisfaction levels of the patients in the PC centre in the health centres in Ankara and the factors affecting this level of satisfaction.	Completely dependent patients and patients who have had previous experience of bed rest have higher satisfaction levels. Better physical conditions of PC clinics, comfortable single patient rooms and having materials that they may need, positively affect patient satisfaction. In addition, nursing care satisfaction levels of nurses with higher education level, nurses with more clinical experience, and PC clinics with higher number of nurses are higher.
Saygılı [342]	Turkish	Thesis	Cross-sectional	To evaluate the cost effectiveness of the PC services received by cancer patients who need PC with three different service models.	Receiving usual care at the public hospital was more cost effective than receiving care at the Palliative Care Centre from the social perspective. From the patient’s perspective, home health care services model was more cost effective than the usual care at the public hospital model and the Palliative Care Centre model. When the gains in terms of quality of life were evaluated from the patient’s perspective, the most cost effective model became the home health care services model again. When the patient satisfaction level was evaluated from the patient’s perspective, the most cost-effective method was found as the usual care at public hospital model. The most cost-effective model in terms of the satisfaction levels and the care burdens of the families was found as home health care services model.
Gürakan [343]	Turkish	Thesis	Interventional	To investigate the impact of aromatherapy massage on pain and plasma beta-endorphin levels in palliative cancer patients.	Aromatherapy massage can decrease pain intensity and increase plasma beta-endorphin levels in palliative cancer patients.
Ahmed *et l* [13]	Turkish	Thesis	Cross-sectional	To comment on the use of narcotic analgesics.	Cancer pain is still one of the most feared entities in cancer and about 75% of cancer patients with pain require treatment with opioids for severe pain.
Akyüz [344]	Turkish	Thesis	Cross-sectional	To investigate if the existence of a PC centre in the hospital makes a difference on the hospital and healthcare giver satisfaction of terminally ill cancer patients.	Having a PC centre in the hospital positively impact hospital and healthcare provider satisfaction of the cancer patients. The patients who are in PC centres do not have an intention to switch to standard hospitals without any PC centres. Additionally they would like to receive psychological support during their treatment process.
Uslu [345]	Turkish	Thesis	Cross-sectional	To determine the PC practices of the midwives and nurses working in the gynaecological oncology units in Ankara.	Hospitals with gynaecological oncology units in Ankara were evaluated and it was suggested to enhance the awareness of midwives and nurses on the PC, include the PC in the graduate curriculum and on-job training programs, organise a number of courses in this field and develop a number of guidelines for symptomatic checks in PC.
Özçelik [346]	Turkish	Thesis	Interventional	To examine the effect of case management model on symptom level, quality of life, patient and family satisfaction, and direct cost results in PC of cancer patients.	In PC of cancer patients, a better symptom control was provided and their life quality was improved (excluding physical and cognitive functions) through the use of Case Management Model. Satisfaction level of patients and families monitored by Case Management is higher.
Turgay [347]	Turkish	Thesis	Cross-sectional	To explore healthcare professionals’ knowledge and views on PC.	53.7% of the health professionals did not receive education on PC. The barriers for development of the PC services in Turkey were listed as ignorance (44.4%) and lack of education (42.9%). PC should be provided in curricula and in-service education programmes to increase awareness among healthcare professionals.
Çakıcı [83]	Turkish	Thesis	Cross-sectional	To evaluate the knowledge of the physicians and nurses for PC and PC practices of them for children with end-stage cancer.	76%–93% of the participants showed correct approach in management of the other symptoms. Only 19% of the participants stated that their abilities in end-stage care were sufficient and 82% of them indicated that they needed training in end-of-life care.
Ekici Kocakafa [348]	Turkish	Thesis	Cross-sectional	To determine the relationship between the socio-demographic characteristics of caregivers and their burden of care.	Increased duration of patient care, lack of social security, increased number of people in need of home care outside the patient, negative effects of family dynamics during the caregiving process, increased weight of patient resulted in an increase in Burden Interview Score.
Karabuğa [349]	Turkish	Thesis	Methodological	To evaluate reliability and validity of The Caregiver Quality of Life Index-Cancer Scale and to investigate quality of life and factors related to quality of life among caregivers of patients with cancer.	The Caregiver Quality of Life Index is valid and reliable. Caregivers who were female, old, had financial difficulties and had difficulties to continue their routine responsibilities or duties and who weren’t supported had lower quality of life scores.
Çalışkan [350]	Turkish	Thesis	Cross-sectional	To determine the efficiency of Algology Department, by assessing the cancer patients, their doctors and nurses working in some of the surgical and oncology departments of Hacettepe University Faculty of Medicine.	61.9% of the patients were suffering from pain. After the follow-up, 88.6% of the patients, 70% of whom were informed about the treatment of cancer pain by the doctors and nurses of Algology Department, mentioned that their complaints had partially or completely passed. 78.8% of nurses had joined the education programmes after graduation which were given by Algology Department. 65% of the doctors were at least seeing a patient once a week suffering from cancer pain but most of them were found not to have adequate education about cancer pain and its treatment during their medical school or residency training.
Elevli [351]	Turkish	Thesis	Cross-sectional	To quantify the dependency ratio of geriatrics group, and to specify the need of home care service by designating the needs assessment.	Home care services, ageing and its problems, population growth of aged people, nursing of aged people, and palliative and hospice care services which are related to terminal period of cancer are discussed. Our country’s need for care services and its current state have been attempted to be determined by examining the examples in Turkey and the world. By considering that cancer patients and geriatric group need home care service, the service area has been attempted to be specified.
Kılınç [352]	Turkish	Thesis	Cross-sectional	To analyse admission rate, prognosis, mortality rates, risk factors affecting mortality and cost per patient of cancer patients followed in Pamukkale University Faculty of Medicine Anestesiology Intensive Care Unit.	The most common reason for admission was respiratory failure (63.3%), followed by sepsis (16.3%) and cardiac arrest (5.17%). Cost per patient per day in ICU was between 186.9-4407.4 TL and mean cost was 1,628.5 ± 524.1 TL. Mortality rate of these cancer patients was 89.2%.
Uzunkaya [353]	Turkish	Thesis	Cross-sectional	To determine attitudes of nurses, working in oncology clinics, towards care of dying patient and principles about death with dignity and their opinions regarding good death.	Nurses’ most important difficulty in giving care to the dying patient was communication with patients and their relatives. Attitudes of nurses towards giving care to the dying patient were more positive (*t* = 4.900; *p* = 0.001). Good death score median of female nurses was higher male nurses (*p* = 0.001). There was a negative weak correlation (*r* = −0,158; *p* = 0.011) between nurses’ level of adopting principles about death with dignity and their perceptions about good death; a positive weak correlation between attitudes of nurses for giving care to the dying patient and psychosocial-spiritual (*r* = 0.124; *p* = 0.011) and self-control subscales (*r* = 0.172; *p* = 0.006) of death.
Terzi [354]	Turkish	Thesis	Qualitative	To determine the views of patients with cancer and their nurses regarding end-of-life care and to contribute to the care needs of patients.	In general, patients and nurses defined end-of-life care as a time when painless death is near, physical and psychological needs increase and team collaboration is absolutely necessary. Although nurses want to provide the necessary care, they are not able to do so due to excess workload.
Yalçın [355]	Turkish	Thesis	Cross-sectional	To evaluate end-stage patients admitted to the Anesthesia Intensive Care Unit, and their intensive care procedures, including PC receiving score and life expectancy according to palliative prognostic index, mortality rates and risk factors affecting mortality, including per patient costs by a prospective analysis.	The most common primary disease for intensive care admission was malignancy. The average cost of treatment for exitus patients was $ 3,654.50, while the cost of treatment for surviving patients was $ 7,053.38. Patients who are dying or refusing treatment due to terminal diseases, determined using the necessary prognostic scoring systems, should decide together with their primer physician and be admitted to a PC unit or hospice.
Özyalçın [356]	Turkish	Thesis	Cross-sectional	To find out about the opinions of cancer patients, their relatives and nurses about death and good-death.	The nurses have defined the death as function interruption physically while the patients and patient relatives have defined the good death as living without suffering. Patients’ views about good death were found to be more positive, fear of death and avoidance behaviors were lower and meaningful. Nurses have more fear of death and avoidance of death. Therefore, comprehensive care should be given to the end-of-life care of nurses.
Şipal [357]	Turkish	Thesis	Cross-sectional	To determine the relationship between supportive care needs and disease acceptance levels of patients with haematological cancer.	The difference between the perception of social support, age, number of cures and outpatient or inpatient treatment and disease acceptance level of the patients were statistically significant. On the other hand, supportive maintenance requirements; sex, marital status, income level, social support perception, presence of chronic disease, metastasis status, outpatient or inpatient treatment had been found to be affecting. When the need for mental and physical care of the patients increased, the acceptance level of the disease decreased.
Palliative Care Association [25]	Turkish	NGO Website	-	The establishment’s purpose was to provide care for patients with cancer and to ensure the establishment and development of PC services across the country.	Palliative Care Association was established in Istanbul on 4-12-2005, The main objective was to support the formation of the necessary structures to provide PC. Association helped to develop cooperation between physicians and healthcare professionals. It also publishes magazines, brochures, bulletins. The member advised the MoH in formulating policies related to PC. Joint projects have been carried out with national and international organisations.
Keskinkılıc *et al* [29]	English	Turkey Cancer Control Programme	-	Structure and Principals of National Cancer Control Programme.	A comprehensive National Cancer Control Programme regarding the cancer registry, early detection, and treatment of the cases, development of treatment guidelines enable symptom control and high quality of life as much as possible for the patients at an advanced stage.
Turkish Ministry of Health [30]	Turkish	Legislation/regulation	-	Quality standards for healthcare training/education and implementation of the evaluation plan.	Training programme for the nurses to acquire the knowledge, skills and attitudes required to provide PC for patients and their families.
The Presidency of the Republic of Turkey [31]	Turkish	The Official Gazette	-	Organisation and job description.	Job descriptions of the General Directorate of Public Health and the General Directorate of Public Hospitals. The duties of the General Directorate of Public Hospitals are; to ensure the execution of all kinds of preventive, diagnosis, treatment and rehabilitative health services in health institutions, and to evaluate the activities, to follow good practice.
General Directorate of Public Hospitals [32]	Turkish	MoH website	-	Procedures and practice related to Home Health Services.	‘Home Health and Palliative Care Unit’ has established under the General Directorate of Public Hospitals Health Services Department.
The Prime Minister’s office of the Republic of Turkey [36]	Turkish	The Official Gazette	-	The scope, basis, definitions and principles of patient rights regulations in Turkey.	The principles and procedures for ensuring that everyone can benefit from patient rights, to be protected from violations of rights, and, when necessary, to use legal safeguards as necessary.
The Presidency of the Republic of Turkey [37]	Turkish	The Official Gazette	-	To regulate the procedures and principles that the institutions and organisations, must comply with to provide home care services.	Continuity of home care is essential and should be available 24 hours including weekends and holidays. The person who wants to receive home care can apply to the health institution in person or by phone. The call centre interviews the person requesting services and provides the necessary information. The person who wants to receive care at home will be visited by a physician and nurse. The care needs of the person and home conditions will be determined and a home care treatment plan will be developed. If necessary, patient consultation will be provided with specialist physicians.
Turkish Ministry of Health [38]	Turkish	Directive	-	Directive on the Implementation Procedures and Principles of Home Health Services Offered by the Ministry of Health.	The Ministry of Health will provide medical care, rehabilitation, social and psychological support services to those individuals and family members who need home health services through establishing home healthcare units within the affiliated health institutions.
Turkish Ministry of Health [39]	Turkish	Directive	-	Applicatıon procedures and prıncıples of PC servıces.	The purpose of this directive is to early identification of pain and other symptoms in patients having life-threatening diseases and to provide medical, psychological, social and moral support to these patients and their family members. To establish PC centres define their functioning and physical conditions, the minimum standard of equipment and personnel. To determine the procedures and principles regarding the duty, authority, and responsibilities of the pallıatıve care servıces.
The Presidency of the Republic of Turkey [40]	Turkish	The Official Gazette	-	The notification for amending the Social Security Institution Healthcare Implementation.	The necessary criteria have been determined for the evaluation of PC treatment within the scope of reimbursement. The first of these criteria is that the relevant health facility must be registered by the Ministry of Health to provide PC.
The Presidency of the Republic of Turkey [41]	Turkish	The Official Gazette	-	This Regulation covers the institutions and organisations that provide home health services and is related to the provision of this service.	The purpose of this Regulation; to determine the procedures and principles for ensuring coordination between institutions and organisations related to the provision, referral and administration of home health services (medical care, rehabilitation, social and psychological support to patients and their family members) provided by the Ministry and its affiliated institutes.
The Presidency of the Republic of Turkey [45]	Turkish	The Official Gazette	-	Regulation for the establishment of Ege University Palliative Care Application and Research Center.	This Regulation covers the provisions regarding the objectives, fields of activity, governing bodies, duties of management bodies and working principles of Ege University Palliative Care Application and Research Center.
Palliative Care Nurses Association [48]	Turkish	NGO Website	-	To increase the professional knowledge and experience in the field of PC, monitor the developments in nursing practices.	The PC nurses association was founded in 2019. The Association organises congresses, conferences, seminars and symposiums related to PC nursing. Cooperates with relevant national and international institutions and organisations. Publishes magazines, brochures. Determines the principles of professional practice, ethical rules, standards of application, in the field of PC. Organises activities for patients, their families and society to improve awareness and quality of life.

**Supplementary Table 2.** Scales used as assessment tool in PC for cancer research in Turkey.

European Organization for Research and Treatment of Cancer Quality of Life Core Questionnaire version 2.0 (EORTC QLQ-C30 v.2.0) [92, 93, 116]
Edmonton Symptom Assessment Scale (ESAS) [94, 97, 116, 117]
Barriers Questionnaire II (BQ-II) [98]
Memorial Symptom Assessment Scale (MSAS) [99, 100]
Beck Hopelessness Scale (BHS) [101]
Beck Depression Scale (BDS) [101–103]
Perceived Social Support From Family Scale (PSS-Fa) [103]
Loneliness Scale (UCLA-LS) [103, 104]
Memorial Symptom Assessment Scale-Short Form (MSAS-SF) [105]
Condensed Memorial Symptom Assessment Scale (CMSAS) [105]
Family Satisfaction Scale (FAMCARE Scale) [106, 116]
European Organization for Research and Treatment of Cancer Quality of Life Questionnaire-Core 15-PAL (EORTC QLQ–C15–PAL) [107]
Three Levels Of Needs Questionnaire (3LNQ Scale) [108]
Posttraumatic Stress Disorder Check-List—Civilian Version (PCL-C) [109]
Cancer Behavior Inventory-Brief Version (CBI-B) [110]
Palliative Performance Scale (PPS) [97, 111, 117]
Palliative Prognostic Index (PPI) [111]
Delirium Rating Scale (DRS) [111]
Functional Assessment of Chronic Illness Therapy–Palliative Care (FACIT-Pal) [112]
The Karnofsky Performance Scale (KPS) [113]
Patient Dignity Inventory (PDI) [118]
The Turkish Version of Palliative Care Outcome Scale (POS) [114]
Patient Safety Culture Scale (PSCS) [115]
Caregiver Quality of Life Index-Cancer Scale (CQOLC) [119]
